# Changes in attitudes toward the HPV vaccine in Kenya

**DOI:** 10.1371/journal.pgph.0007508

**Published:** 2026-10-23

**Authors:** Corrina Moucheraud, Jinghong Miao, Eric Ochieng, Suyanpeng Zhang, George Ganda, Ginger Golub, Risa M. Hoffman, Peter G. Szilagyi, Sze-Chuan Suen

**Affiliations:** 1 Department of Public Health Policy and Management, New York University School of Global Public Health, New York, New York, United States of America; 2 Daniel J. Epstein Department of Industrial and Systems Engineering, University of Southern California Viterbi School of Engineering, Los Angeles, California, United States of America; 3 Innovations for Poverty Action, Sandalwood Lane, Nairobi, Kenya; 4 Department of Medicine, University of California Los Angeles David Geffen School of Medicine, Los Angeles, California, United States of America; 5 Department of Pediatrics, University of California Los Angeles David Geffen School of Medicine, Los Angeles, California, United States of America; Hamadan University of Medical Sciences School of Public Health, IRAN, ISLAMIC REPUBLIC OF

## Abstract

Globally, attitudes about vaccines and vaccination are changing rapidly, but few studies have examined within-person shifts over time. We use a longitudinal survey in Kenya to assess how respondents’ attitudes about the human papillomavirus (HPV) vaccine changed between 2022 and 2024. In 2022, we conducted a random digit-dialed phone survey of 1405 Kenyan parents/caregivers of girls age-eligible for the HPV vaccine; respondents were re-contacted in 2024, and 1093 people (78%) were successfully re-surveyed. Each survey included identical questions about HPV vaccine and vaccination attitudes (adapted from the Vaccine Hesitancy Scale-HPV). We estimate direction and degree of each person’s change in attitude, overall and by construct/question, and explore associations between these changes and respondent demographics, and baseline knowledge about HPV and HPV vaccination. Attitudes about the HPV vaccine changed substantially from 2022 to 2024. Although perceptions of the vaccine’s importance and effectiveness were relatively stable (only 25% of respondents reported any attitude change), there were significantly more changes among respondents with lower educational attainment and who were unemployed. Safety perceptions changed substantially over this period: two-thirds of respondents changed their beliefs around the vaccine’s side effects and riskiness. Approximately one-quarter of respondents developed a worse safety attitudes, while 18.9% of respondents improved their safety attitude. Among individuals with lower socioeconomic status, attitudes about following a clinician’s HPV vaccination recommendation and riskiness of the HPV vaccine significantly worsened over this period. We found that, in this population of Kenyan adults and over a two-year period, attitudes about the HPV vaccine have changed both positively and negatively. Targeted interventions may be needed, particularly with less socioeconomically-advantaged groups, to reinforce messages about the vaccine’s importance, effectiveness, and safety. Further research is needed about reasons for the large changes in safety perceptions, both toward more and less favorable views.

## Introduction

Vaccine attitudes are changing globally. Even before the COVID-19 pandemic, the World Health Organization named “vaccine hesitancy” as a top-10 threat to public health in 2019 [1], and evidence suggests that attitudes about vaccine have continued to worsen after the pandemic [2–6]. Additionally, new vaccines may face unique concerns [7–9] including about side effects and safety, which are important to understand; and new vaccines often receive a surge of public health attention (for example, communication campaigns) so people’s attitudes may change quickly as they are exposed to new information and messages.

Many low- and middle-income countries (LMICs) face a high burden of cervical cancer so HPV vaccination is extremely important [10,11] – but uptake of the HPV vaccine is low in many of these countries [12,13]. Studies have begun to identify attitudinal factors that may explain this low uptake [14–17]. It is important to understand how people’s attitudes about HPV vaccination may be evolving as the vaccine is rolled out (alongside promotion campaigns) in LMICs [18,19]. Kenya introduced the HPV vaccine in 2019 but uptake is low: in 2022, 17% of eligible girls had completed the HPV vaccine series, and this rose to 32% in 2023 and 36% in 2024 [12,14,20]. Many Kenyan parents express concerns about the HPV vaccine including its short- and long-term safety [15,20–22].

There are several reasons why it is important to understand people’s changing attitudes about vaccines. First, studies have identified an overall increase in vaccine hesitancy [2–6], but few have been longitudinal so have not assessed changes at the individual level. Second, by understanding such within-person changes, we can better design effective vaccination promotion programs. For example, we can generate more effective messages for dissemination by key stakeholders who have repeated contacts with eligible young people and their parents/caregivers – such as clinicians, community health workers, or teachers. Relatedly, we can better understand whether and how attitudes change can help inform ongoing vaccination discussions; many experts recommend that health workers engage in shared decision-making over time with parents/caregivers who initially decline the HPV vaccine [23–25], so evidence about which attitudes are most susceptible to change and among whom can help target these conversations.

The objective of this analysis was to explore intra-personal changes over approximately two years in attitudes toward the HPV vaccine among parents/caregivers of girls eligible for the HPV vaccine in Kenya. Leveraging a unique dataset that surveyed respondents in 2022 and again in 2024, we investigate the degree and types of attitudinal changes that occurred in the sample over this period, as well as explore factors hypothesized to be correlated with these changes.

## Materials and methods

In 2022, our team conducted a telephone survey of randomly-selected Kenyan adults who were parents or caregivers (herein called “caregivers”) of girls presently or recently age-eligible for HPV vaccination (i.e., 10–16 years old). Detailed methods have been previously published [14]. In 2024, we conducted a follow-up telephone survey with those respondents who completed the 2022 survey and agreed to be contacted for future studies. This analysis uses data from respondents who participated in both the 2022 and 2024 surveys. Our team’s prior work has identified minimal loss to follow-up and of differential attrition, in this sample [26].

### Survey tool

The 2022 survey tool was developed to reflect domains of the Behavioral and Social Drivers of Vaccination framework [27,28]: what people think and feel about the HPV vaccine, social factors that can influence people’s interest in the HPV vaccine, and access to HPV vaccination services. The specific survey questions leveraged existing, previously-validated surveys whenever possible. The constructs used in this analysis are shown in Table 1; specific questions can be found in S2 Table for attitudes, and S4 Table for knowledge. The 2024 survey replicated the 2022 survey questions about HPV vaccine attitudes and respondent demographics.

**Table 1:** Constructs, survey questions, and operationalization for this analysis.

Construct	Source & adaptations	Operationalization for analysis
HPV vaccine attitudes - Measured in 2022 and 2024	Adaptation of the Vaccine Hesitancy Scale-HPV [29]; chose items that could be well-translated; added detail on side effect severity and duration [30]	Seven items with four-point Likert scale (each could range 1–4)
HPV vaccine knowledge - Measured in 2022	Adaptation of knowledge assessments from previous studies [31,32]	Seven items classified as correct or incorrect, with missing or “don’t know” treated as incorrect (could range 0–1); and total score (sum of items) (could range 0–7)

### Data collection

Using a randomization algorithm, between 1 August 2022–9 September 2022, we selected numbers to call from a list of 25,000 randomly-generated mobile phone numbers. If the call was answered, a trained research assistant asked if the person was interested in hearing about the study and assessed for eligibility, if interested. Eligible respondents were those who identified as a parent or caregiver for a girl aged 10–16 years (i.e., girls who were currently or recently age-eligible for HPV vaccination in Kenya) and those who reported being aware of their daughter’s HPV vaccination status. If eligible, per the protocol approved by Institutional Review Boards (see Ethical Review section), participants provided oral informed consent before beginning the survey; consent to participate in the survey (and to be contacted for future studies) was documented electronically by the research assistant for each potential respondent and only those who provided consent to participate moved on to the survey questions. The survey was then read aloud by the research assistant who entered the responses into data collection software. At the completion of the survey, each respondent received the equivalent of approximately US$ 1.

In total, 19,688 phones were dialed and 10,096 were caregivers who were further screened for eligibility, of whom 1454 were eligible, 1416 agreed to the survey, and 1405 consented to be followed-up for future surveys. From 5 June to 7 October 2024, these same 1405 caregivers were re-contacted and re-consented, and we successfully reached 1158, and 1093 completed the re-survey and constitute the analytic sample for this analysis (77% of those who agreed to be re-contacted, and 94.4% of those who were successfully reached).

### Data analysis

Vaccine attitudes were measured using the Vaccine Hesitancy Scale-HPV [29] (see Table 1): each respondent was asked an identical set of seven questions measuring their attitudes about the HPV vaccine in 2022 and in 2024. Their response to each question on each survey was operationalized using a four-point Likert scale (from more negative to more positive attitudes).

Respondents were matched from the 2022 and 2024 surveys using a unique identifier, and we characterized their attitude changes (by item and overall) by directionality comparing responses in 2024–2022: did they respond more negatively, more positively, identically (no change), or did they shift from “don’t know” to a different response (or vice-versa). We consider “no change” to be the exact same response (or the exact same score) at the two time points. We explore the direction, and magnitude, of change for all respondents.

We then explore patterns of directionality of attitudinal change by respondent characteristics, i.e., gender (female, or male), educational attainment (no schooling or less than primary completion, completed primary school, completed secondary school, or beyond secondary school), employment status (unemployed, formally employed, or self-employed/informally employed), marital status (single, married/partnered, previously married [separated, divorced, widowed]), recent household income (insufficient to meet expenses, just sufficient to meet expenses, or more than sufficient to meet expenses), residence area (urban [city], town [trading center], or rural [village]), and religion (Anglican, Catholic, Muslim, Protestant/Pentecostal, Seventh Day Adventist, Traditional/Tribal religion, or not religious). Previous research has shown that these factors are associated with attitudes toward, and uptake of, vaccination in Kenya [14,21,22], and could inform targeting of interventions and programs. We similarly assessed whether there was a significant association between direction of attitude change and a respondent’s baseline knowledge about HPV vaccination (see definitions and operationalizations in Table 1). We assessed whether the direction of attitudinal change was different across these subgroups using a chi-squared test using a p < 0.05 level of significance.

In addition, we fitted multivariable multinomial logistic regression models to assess these associations after adjustment for other respondent characteristics. For each HPV vaccination attitude item, the outcome was categorized as negative change (-1), no change (0), or positive change (+1) between survey waves, with no change serving as the reference category. Covariates included demographic characteristics and HPV knowledge score, defined as the number of HPV-related knowledge questions answered correctly. The HPV knowledge scores were categorized into three levels: low (0–3 points), medium (4–5 points), and high (6–7 points). For categorical covariates, the category with the largest number of observations was used as the reference group. Adjusted odds ratios and 95% confidence intervals were estimated to quantify associations between respondent characteristics and the direction of attitudinal change.

### Ethical review

The 2022 survey was reviewed and approved by the University of California Los Angeles Institutional Review Board (#22-000005), and the Kenya Medical Research Institute Scientific and Ethics Review Unit (#SERU4456), and the 2024 survey was approved by both entities as an amendment to these original protocols.

## Results

Characteristics of the 1093 caregivers who participated in both surveys can be found in Table 2. Just over half of respondents were female (52.5%). Most respondents completed secondary or at least some higher education (31.3% and 44.6% respectively), and most were employed yet household income over the past 12 months was reported as insufficient for 62.0% of respondents. Approximately three-quarters (78.4%) of respondents were married or partnered/cohabitating. 27.7% of respondents (n = 303) lived in urban areas (cities), 29.6% (n = 323) resided in towns (trading centers), and 42.7% (n = 467) lived in rural villages. Approximately half of respondents were Protestant or Pentecostal (48.5%), 22.9% were Catholic, 10.5% were Anglican, 7.2% were Seventh Day Adventist, and 5.3% were Muslim. Very few respondents (1.2% of the sample) were not religious.

**Table 2:** Characteristics of the sample (n = 1,093).

		n	Percent
**Gender**	Male	519	47.5
	Female	574	52.5
**Educational attainment**	No schooling or less than primary	29	2.7
	Completed primary	235	21.5
	Completed secondary	342	31.3
	Beyond secondary (higher education)	487	44.6
**Employment status**	Unemployed	117	10.7
	Formally employed	359	32.8
	Self-employed or informally employed (i.e., casual laborer, subsistence farmer)	614	56.2
	Unknown	3	0.3
**Marital status**	Single	143	13.1
	Married/partnered	857	78.4
	Previously married (separated, divorced, widowed)	84	7.7
	Missing	9	0.8
**Household income over the past 12 months**	Insufficient to meet expenses	678	62.0
	Just sufficient to meet expenses	266	24.3
	More than sufficient to meet expenses	138	12.6
	Missing	11	1
**Residence area**	Urban (city)	303	27.7
	Town (trading center)	323	29.6
	Rural (village)	467	42.7
**Religion**	Anglican	115	10.5
	Catholic	250	22.9
	Muslim	58	5.3
	Protestant or Pentecostal	530	48.5
	Seventh Day Adventist	79	7.2
	Traditional or tribal religion	30	2.7
	Not religious	13	1.2
	Missing	18	1.6

### Which attitudes changed?

The overall distribution of attitudes at both time points are presented in S1 Table, providing context for the individual-level changes described below. Over the two-year follow-up period, 76.2% of caregivers maintained their same attitude about the importance of the HPV vaccine, 76.2% maintained their attitude about the HPV vaccine’s effectiveness, and 80.9% had no change in their stated likelihood of following their clinician’s recommendation for HPV vaccination (Fig 1, S2 Table).

**Fig 1:**
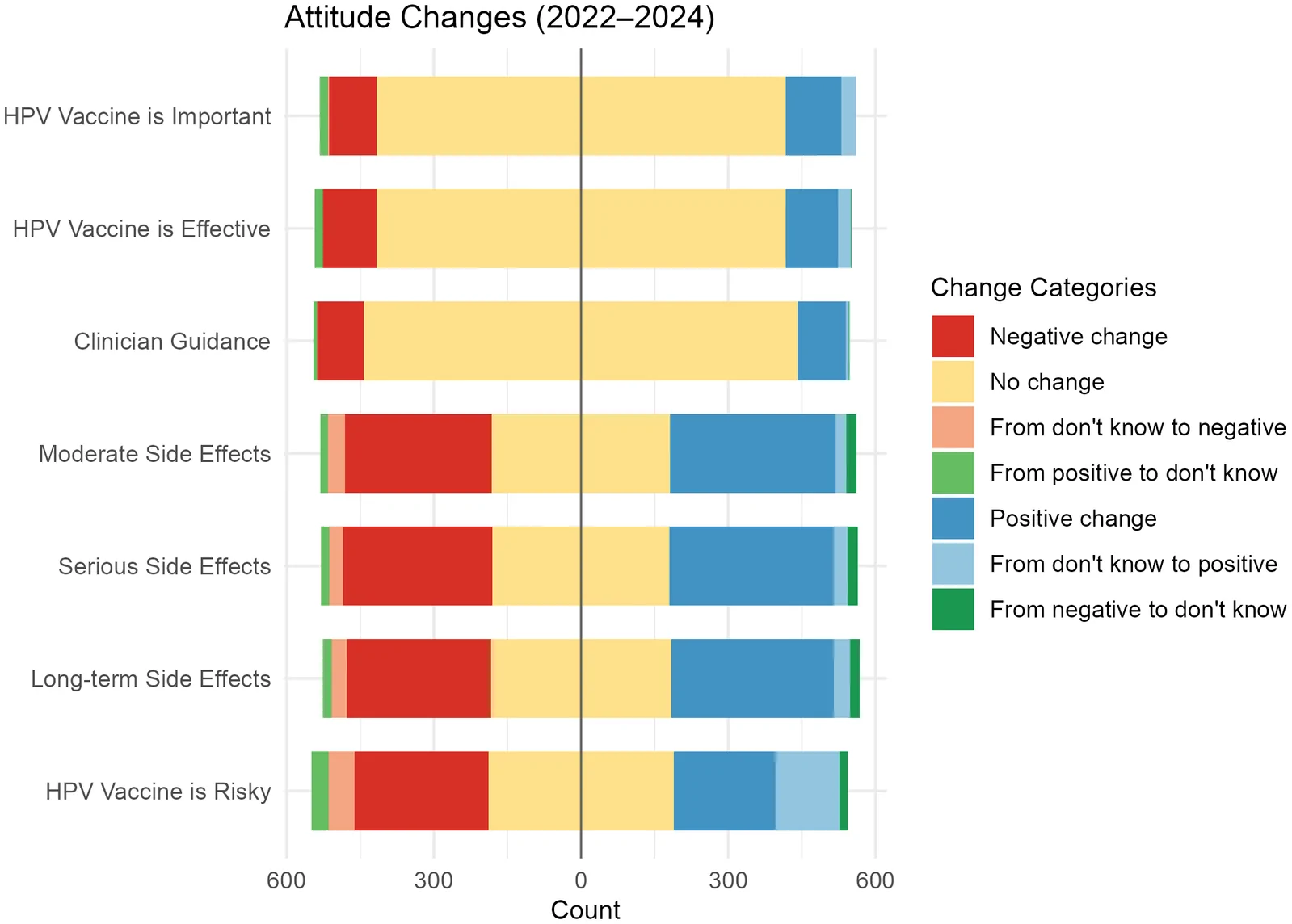
Changes in HPV vaccination attitudes (2022 to 2024).

Larger shifts were seen for attitudes about the HPV vaccine’s safety (Fig 1), and in both positive and negative directions. Approximately two-thirds of respondents changed their beliefs around the vaccine’s mild to moderate short-term effects, serious short-term effects, long-term effects, and whether the HPV vaccine carries more risks than older vaccines, over this period (S2 Table). 730 caregivers (66.8% of respondents) changed their mind about mild to moderate short-term side effects from the HPV vaccine. Of these, 30.8% was a shift toward more positive attitudes (n = 337), and 27.4% (n = 300) was a shift toward more negative attitudes (S2 Table). Caregivers who changed their mind about serious short-term side effects or long-term side effects—67.0% and 66.4% of each group, respectively—were about equally likely to have shifts of smaller or larger magnitudes (S2 Table). For all questions there were also some caregivers who changed to or from answering “I don’t know” (both toward more positive and negative sentiments); the question with the most shift was, “The HPV vaccine carries more risks than older vaccines”: 131 respondents who said “I don’t know” in 2022 changed to a favorable attitude by 2024 (12.0%), 52 changed from “I don’t know” to a negative attitude (4.8%), 35 changed from a positive attitude to “I don’t know” (3.2%), and 27 changed from a negative attitude to “I don’t know” (1.6%). For all other questions, fewer than approximately 3% of respondents switched to or from “I don’t know.”

### Whose attitudes changed?

Caregivers with no or less than primary schooling had a significantly more negative shift in some attitudes—including whether they would follow a clinician’s recommendation about HPV vaccine (17.2% felt more negatively about this in 2024 than in 2022 while 6.9% felt more positively, p-value = 0.016) and that the HPV vaccine is riskier than other vaccines (41.4% felt it was riskier in 2024 than in 2022 versus 20.7% felt it was less risky, p-value = 0.008)—than respondents with greater educational attainment (Fig 2, S3 Table). A similar significant pattern was seen among unemployed respondents who had significantly more negative attitudes for these items in 2024 than in 2022 compared to their employed peers. Relatively more caregivers in urban areas felt worse about following a clinician’s recommendation about HPV vaccination in 2024 than in 2022 (13.2% of urban caregivers with a worse attitude in 2024 for following a clinician recommendation compared to 2022, versus 6.2% of respondents in towns with a worse attitude about following a clinician recommendation in this period, and 7.7% in rural areas, p < 0.001). All attitude changes by all respondent characteristics can be seen in S3 Table.

**Fig 2:**
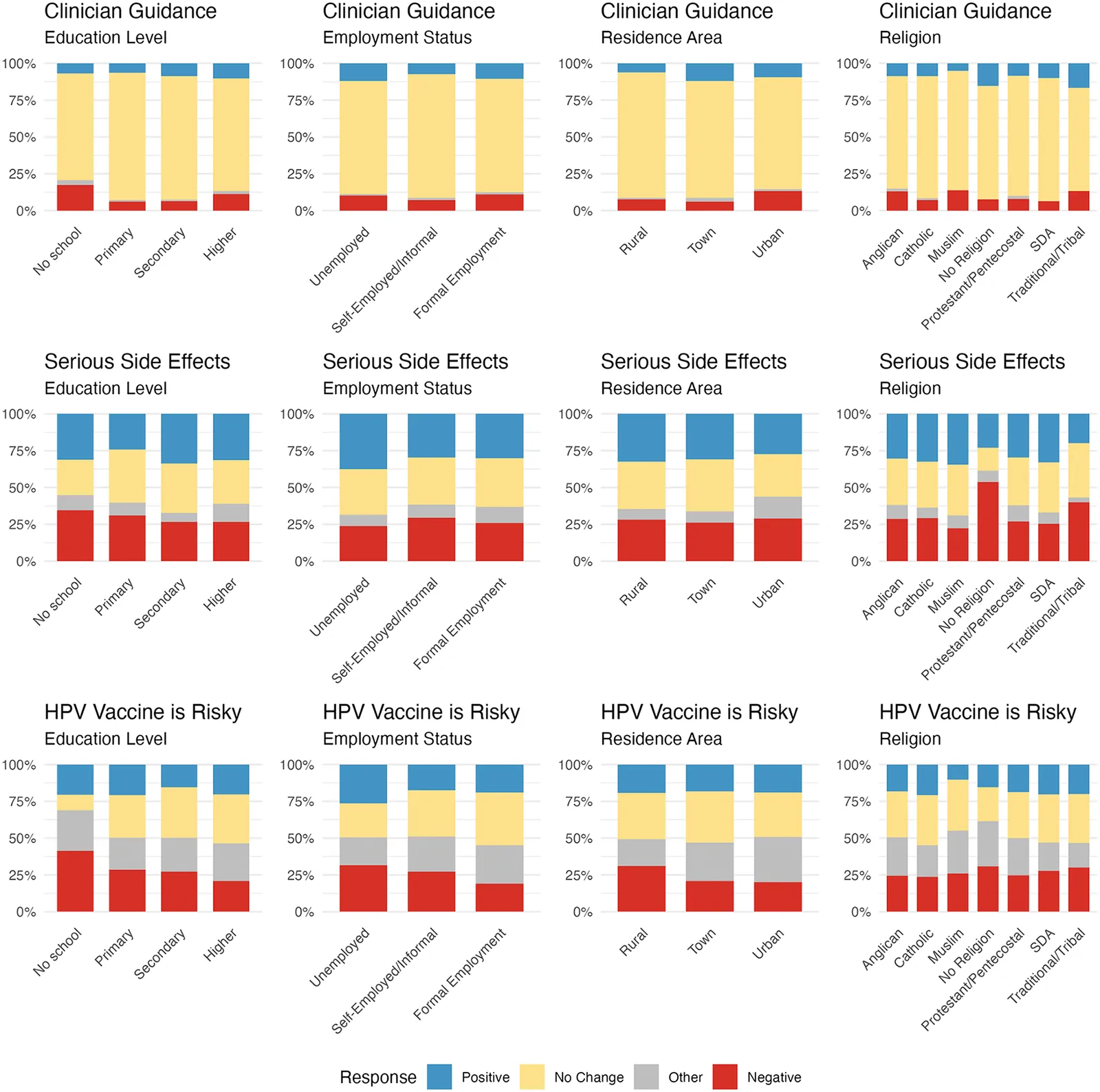
Selected attitude changes by respondent demographics (2022 to 2024).

Overall knowledge at baseline (summary score) was not associated with changing one’s attitudes (Fig 3, S4 Table) including in multivariable models that included respondent characteristics (S8 Table) – but there was a significant association between some questions around the type of knowledge about HPV and changed attitudes. Caregivers who knew that HPV is sexually transmitted were significantly less likely to develop a more negative view about the HPV vaccine’s effectiveness from 2022 to 2024 than those who did not know this (7.8% versus 12.9%; p = 0.004) (S4 and S6 Tables). Respondents who knew that men could get HPV were significantly more likely to report increased concern about the riskiness of the HPV vaccine than people who did not know this (29.3% versus 22.1% respectively; p = 0.027). Caregivers who knew that the HPV vaccine is most effective when given before sexual debut were significantly less likely to develop a more negative view of its protective benefits: 8.4% of respondents who knew this had a worse attitude in 2024 than in 2022, versus 14.7% of those who did not know this (p = 0.001); and were significantly more likely to report an increased concern about its risks in 2024 than in 2022 compared to those who didn’t know this (17.8% versus 27.5%; p = 0.017).

**Fig 3:**
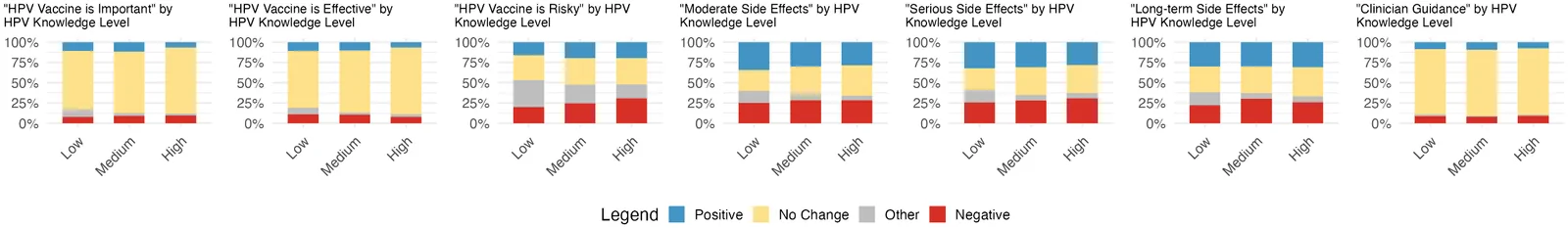
Attitude changes (2022 to 2024) by respondent’s baseline HPV knowledge.

We find that the multivariate analyses identified statistically significant associations with change in attitudes in urbanicity and religion (traditional/tribal affiliations and Seventh Day Adventists), suggesting that these factors may play a role in attitude change over time. Specifically, respondents living in towns had higher odds of a positive attitude change about following clinician guidance, and those residing in urban areas had higher odds of disagreeing more that HPV vaccine is riskier than other vaccines. Individuals identifying with traditional or tribal religions were more likely to exhibit negative shifts in attitude regarding long-term side effects, indicating increased belief that the vaccine has long-term side effects. Detailed results are provided in the supplement S8 Table.

## Discussion

The HPV vaccine is a relative newcomer to the immunization space, having been introduced to many countries’ national programs within the past decade; Kenya introduced the vaccine in 2019, for example. Its emergence has thus coincided with worsening attitudes about vaccination globally. It is also a unique vaccine in countries where vaccination after early childhood is rare. In this examination of within-person changes in attitudes toward the HPV vaccine over two years in Kenya, we found that attitudes about the vaccine’s importance and effectiveness were more stable than attitudes about safety; and that people were changing their minds – particularly about the vaccine’s safety – in both positive and negative directions. Our study highlights that while perceptions about HPV vaccination remained stable for many people in this sample, a substantial subgroup of people developed greater concerns about vaccine safety despite a preponderance of data demonstrating the short- and long-term safety of the HPV vaccine [33,34]. To our knowledge this is the first within-person analysis that has explored attitudes toward HPV vaccination, with some caregivers being more positive and others being more negative.

Over three-quarters of respondents maintained their same positive attitude about the HPV vaccine’s importance and effectiveness. Our team’s previous work has described the very widespread endorsement of these beliefs in this sample, with over 95% of respondents agreeing that the HPV vaccine is important for their daughter’s health and that the HPV vaccine is a good way to protect their daughter from disease [14]. Taken together, these findings indicate that overall, this randomly-selected population of Kenyan parents/caregivers of preadolescent girls had positive and stable attitudes about importance and effectiveness of the HPV vaccine.

However, approximately two-thirds of caregivers in this study changed their attitude about the HPV vaccine’s side effects and riskiness over this period, and this was almost evenly split between those whose feelings became more positive and more negative. Analyses of the National Immunization Survey-Teen in the United States has found an increase in parents citing safety concerns as a reason for HPV vaccine hesitancy [35] – but our survey included multiple dimensions of safety (three different types of side effects and a measure of riskiness) and did not ask parents/caregivers to attribute this to vaccine hesitancy so may represent a more nuanced measurement of these attitudinal changes. That we see the same type of prevalent and symmetric attitudinal shifts across our measures of HPV vaccine safety strengthens our confidence in this result.

Although we did not find an overall association between baseline HPV knowledge and attitudinal shifts, caregivers who knew that HPV is sexually transmitted and that girls should be vaccinated before they are sexually active experienced fewer negative changes in their attitudes about the vaccine’s importance and effectiveness over this period. There is considerable interest in identifying ways to “inoculate” people (make them less vulnerable to) misinformation [36,37], and these results suggest interesting opportunities to investigate this concept. Although an educational intervention may not be sufficient by itself to shift vaccination behavior, given the myriad and complex factors that affect vaccination decision-making, it can play an important role [38].

Health worker recommendation is one of the most powerful interventions to increase vaccination [39,40], and we find that over 80% of respondents have no change in their stated intention to follow a health worker’s recommendation for HPV vaccination. However, negative changes – i.e., less willingness to follow a health worker’s recommendation in 2024 than in 2022 – were significantly more common among respondents with low educational attainment. Therefore, if we implement health worker recommendation as a strategy to increase HPV vaccination in Kenya or similar settings like Kenya, it would be important to understand why this intervention may be losing its resonance among certain groups, or to identify successful messengers for reaching certain groups. Future research should seek to explore this phenomenon.

We note that a recent multi-country study in Africa that used repeated cross-sectional surveys found worse attitudes in 2022 than in 2020 among Kenyans about the general importance of vaccines for children and for adults, and better attitudes overall about vaccines’ effectiveness and safety [3]. This divergence with our results may be due to differing time periods, our focus on HPV vaccine only, and differing samples. We encourage further research on this topic – that asks about a greater range of vaccines including, specifically, vaccines given outside the routine childhood schedule such as the HPV vaccine – to deepen our understanding of these attitudinal changes.

While the characteristics associated with changing attitudes may vary across settings or vaccines, our findings about the evolution of people’s attitudes has several potential implications for clinical practice and public health. First, in their communication about vaccination, clinicians should address common concerns and leverage specific techniques for discussing vaccination with individuals who are hesitant. Multiple studies have demonstrated that training in vaccine communication, including incorporating strategies from motivational interviewing, can address hesitancy and improve HPV vaccination rates [41–46]. Additionally, because attitudes can shift over time, repeated discussions may be needed and each conversation should be tailored to current concerns. One way for a clinician to set up the expectation of such conversations at preventive visits is by reminding parents that the issue of vaccination will be raised again at the next visit because they care about the child’s heath [47–49].

This study has numerous strengths, including its use of random-digit dialing to constitute the study sample, its longitudinal design that allows analysis of within-person changes over time, a high rate of retention for the follow-up survey, and its robust measurement of HPV vaccine attitudes using validated instruments. Some limitations should also be noted. First, the Vaccine Hesitancy Scale-HPV (used to measure HPV vaccine attitudes here) was developed and validated in the United States; we made adjustments to the scale for its use in Kenya and pilot tested it before data collection, but further analysis is needed on the psychometric properties of this scale in settings like Kenya to determine its reliability and validity as a measure in low- and middle-income countries. Second, due to the sample construction (i.e., parents/caregivers of preadolescent girls) we had limited variation for certain characteristics that may be more strongly associated with changing attitudes like age [2]. Third, knowledge was only measured in 2022 so we were unable to assess whether these changed over time, and consequently whether knowledge changes were associated with changes in attitudes.

## Conclusions

In this study of Kenyan caregivers of girls age-eligible for HPV vaccination, we find relatively substantial shifts in attitudes about the HPV vaccine’s safety – moving in both more positive and more negative directions – between 2022 and 2024. This suggests that interventions to provide updated information about the vaccine’s side effects and safety profile are continually needed and will have to address two diverging subgroups of people: those with growing support for vaccination, and those with growing concerns. In addition, although attitudes about the vaccine’s importance and effectiveness were overall quite stable over this period, reinforcement of these messages to certain groups (e.g., people with lower educational attainment) may be needed in order to foster positive attitudes about the HPV vaccine and ultimately contribute to the vaccine’s uptake.

## Supporting information

S1 Table Distribution of HPV vaccine attitude responses in 2022 and 2024, presented as count (percentage). (DOCX)

S2 Table Changes in attitude between 2022 and 2024, by degree. (DOCX)

S3 Table p-values of chi-square tests of any attitude change by demographic variables. (DOCX)

S4 Table p-values of chi-square tests of a negative attitude change by knowledge (about cervical cancer, HPV, and HPV vaccination). (DOCX)

S5 Table Baseline attitudes (2022) by demographic and by level of knowledge (about cervical cancer, HPV, and HPV vaccination). (DOCX)

S6 Table Attitude changes by (2022–2024) by demographic and by level of knowledge (about cervical cancer, HPV, and HPV vaccination). (DOCX)

S7 Table Transitions to and from “I don’t know” responses between 2022 and 2024, with percentages calculated within the “I don’t know” population at each time point. (DOCX)

S8 Table Odds ratios from multivariate logistic regression analyses. Values greater than 1 indicate higher odds of a positive attitude change, whereas values less than 1 indicates higher odds of a negative attitude change. Statistically significant estimates (p < 0.05) are marked with an asterisk.
(DOCX)

S1 Checklist Questionnaire on inclusivity in global research. (DOCX)
