# Supplementary material for: Changes in attitudes toward the HPV vaccine in Kenya
Source: PLOS Glob Public Health. 2026 Oct 23;6(10):e0007508. doi: 10.1371/journal.pgph.0007508 (PMC13693870; doi:10.1371/journal.pgph.0007508)
Supplement: Supplementary file 1 [file pgph.0007508.s001.docx]

S1 Table: Distribution of HPV vaccine attitude responses in 2022 and 2024, presented as count (percentage).

| Question | Year | Strongly agree | Somewhat agree | Somewhat disagree | Strongly disagree | Don't know |
| --- | --- | --- | --- | --- | --- | --- |
| The HPV vaccine is important for my daughter’s health | 2022 | 917 (83.9%) | 98 (9.0%) | 16 (1.5%) | 29 (2.7%) | 33 (3.0%) |
|  | 2024 | 938 (85.8%) | 116 (10.6%) | 7 (0.6%) | 12 (1.1%) | 20 (1.8%) |
| Getting the HPV vaccine is a good way to protect my daughter from disease | 2022 | 925 (84.6%) | 99 (9.1%) | 16 (1.5%) | 25 (2.3%) | 28 (2.6%) |
|  | 2024 | 923 (84.4%) | 131 (12.0%) | 8 (0.7%) | 12 (1.1%) | 19 (1.7%) |
| I do what the clinician recommends about HPV vaccine | 2022 | 969 (88.7%) | 82 (7.5%) | 14 (1.3%) | 21 (1.9%) | 7 (0.6%) |
|  | 2024 | 973 (89.0%) | 80 (7.3%) | 10 (0.9%) | 21 (1.9%) | 9 (0.8%) |
| The HPV vaccine carries more risks than older vaccines | 2022 | 152 (13.9%) | 88 (8.1%) | 89 (8.1%) | 553 (50.6%) | 211 (19.3%) |
|  | 2024 | 177 (16.2%) | 138 (12.6%) | 169 (15.5%) | 529 (48.4%) | 80 (7.3%) |
| I am concerned about mild to moderate short-term effects of HPV vaccine | 2022 | 344 (31.5%) | 172 (15.7%) | 85 (7.8%) | 429 (39.2%) | 63 (5.8%) |
|  | 2024 | 250 (22.9%) | 255 (23.3%) | 149 (13.6%) | 395 (36.1%) | 44 (4.0%) |
| I am concerned about serious short-term effects of HPV vaccine | 2022 | 317 (29.0%) | 167 (15.3%) | 89 (8.1%) | 453 (41.4%) | 67 (6.1%) |
|  | 2024 | 239 (21.9%) | 233 (21.3%) | 141 (12.9%) | 435 (39.8%) | 45 (4.1%) |
| I am concerned about long-term effects of HPV vaccine | 2022 | 347 (31.7%) | 153 (14.0%) | 80 (7.3%) | 444 (40.6%) | 69 (6.3%) |
|  | 2024 | 261 (23.9%) | 209 (19.1%) | 138 (12.6%) | 443 (40.5%) | 42 (3.8%) |
