# Supplementary material for: Changes in attitudes toward the HPV vaccine in Kenya
Source: PLOS Glob Public Health. 2026 Oct 23;6(10):e0007508. doi: 10.1371/journal.pgph.0007508 (PMC13693870; doi:10.1371/journal.pgph.0007508)
Supplement: Supplementary file 2 [file pgph.0007508.s002.docx]

S2 Table: Changes in attitude between 2022 and 2024, by degree

|  | No change | Positive change | | | From DK to positive | Negative change | | | From DK to negative | From negative to DK | From positive to DK |
| --- | --- | --- | --- | --- | --- | --- | --- | --- | --- | --- | --- |
|  |  | +1 | +2 | +3 |  | -1 | -2 | -3 |  |  |  |
| The HPV vaccine is important for my daughter’s health | 833 (76.2%) | 78 (7.1%) | 17 (1.6%) | 19 (1.7%) | 29 (2.7%) | 87 (8.0%) | 7 (0.6%) | 3 (0.3%) | 2 (0.2%) | 1 (0.1%) | 17 (1.6%) |
| Getting the HPV vaccine is a good way to protect my daughter from disease | 833 (76.2%) | 75 (6.9%) | 15 (1.4%) | 17 (1.6%) | 26 (2.4%) | 96 (8.8%) | 10 (0.9%) | 4 (0.4%) | 0 | 1 (0.1%) | 16 (1.5%) |
| I do what the clinician recommends about HPV vaccine | 884 (80.9%) | 67 (6.1%) | 12 (1.1%) | 18 (1.7%) | 7 (0.6%) | 71 (6.5%) | 9 (0.8%) | 16 (1.5%) | 0 | 1 (0.1%) | 8 (0.7%) |
| The HPV vaccine carries more risks than older vaccines | 378 (34.6%) | 73 (6.7%) | 64 (5.99%) | 70 (6.4%) | 131 (12.0%) | 102 (9.3%) | 77 (7.0%) | 94 (8.6%) | 52 (4.8%) | 27 (1.6%) | 35 (3.2%) |
| I am concerned about mild to moderate short-term effects of HPV vaccine | 363 (33.2%) | 141 (12.9%) | 99 (9.0%) | 97 (8.9%) | 22 (2.1%) | 126 (11.5%) | 96 (8.8%) | 78 (7.1%) | 34 (3.1%) | 21 (1.9%) | 16 (1.5%) |
| I am concerned about serious short-term effects of HPV vaccine | 361 (33.0%) | 118 (10.8%) | 107 (9.8%) | 109 (10.0%) | 29 (2.7%) | 110 (10.0%) | 103 (9.4%) | 91 (8.3%) | 29 (2.7%) | 20 (1.8%) | 16 (1.5%) |
| I am concerned about long-term effects of HPV vaccine | 367 (33.6%) | 102 (9.3%) | 110 (10.0%) | 120 (11.0%) | 33 (3.0%) | 117 (10.7%) | 78 (7.1%) | 98 (9.0%) | 31 (2.8%) | 19 (1.7%) | 16 (1.6%) |
