# Supplementary material for: Changes in attitudes toward the HPV vaccine in Kenya
Source: PLOS Glob Public Health. 2026 Oct 23;6(10):e0007508. doi: 10.1371/journal.pgph.0007508 (PMC13693870; doi:10.1371/journal.pgph.0007508)
Supplement: Supplementary file 3 [file pgph.0007508.s003.docx]

S3 Table: p-values of chi-square tests of any attitude change by demographic variables

| Variable | HPV vaccine is important | HPV vaccine is effective | Do what clinician recommends about HPV vaccination | Concerned about mild to moderate short-term side effects of HPV vaccination | Concerned about serious short-term side effects of HPV vaccination | Concerned about long-term side effects of HPV vaccination | The HPV vaccine carries more risks than older vaccines |
| --- | --- | --- | --- | --- | --- | --- | --- |
| Gender | 0.67441 | 0.18391 | 0.42216 | 0.37506 | 0.22247 | 0.5446 | 0.3638 |
| Education Level | **0.00667** | **0.00787** | **0.01581** | 0.34335 | 0.24162 | 0.35821 | **0.00849** |
| Residence Area | 0.34798 | 0.11665 | **0.00062** | 0.22761 | 0.58153 | 0.71392 | 0.05871 |
| Religion | NA | 0.74869 | 0.5151 | 0.18166 | 0.70473 | 0.18676 | 0.98575 |
| Marital Status | 0.32873 | 0.34303 | 0.76438 | 0.07342 | 0.74792 | 0.39384 | **0.03308** |
| Employment Status | 0.3848 | 0.26991 | 0.04621 | 0.75119 | 0.43141 | 0.6922 | **0.0024** |
| Household Income | 0.38602 | 0.08447 | 0.13488 | 0.54711 | 0.32043 | 0.1533 | 0.81368 |
