# Supplementary material for: Changes in attitudes toward the HPV vaccine in Kenya
Source: PLOS Glob Public Health. 2026 Oct 23;6(10):e0007508. doi: 10.1371/journal.pgph.0007508 (PMC13693870; doi:10.1371/journal.pgph.0007508)
Supplement: Supplementary file 4 [file pgph.0007508.s004.docx]

S4 Table: p-values of chi-square tests of a negative attitude change by knowledge (about cervical cancer, HPV, and HPV vaccination)

| Variable | HPV vaccine is important | HPV vaccine is effective | Do what clinician recommends about HPV vaccination | Concerned about mild to moderate short-term side effects of HPV vaccination | Concerned about serious short-term side effects of HPV vaccination | Concerned about long-term side effects of HPV vaccination | The HPV vaccine carries more risks than older vaccines |
| --- | --- | --- | --- | --- | --- | --- | --- |
| HPV can cause cervical cancer | 0.96381 | 0.14695 | 0.87326 | 0.18362 | 0.22054 | 0.41042 | 0.7991 |
| HPV can be passed on during sexual intercourse | 0.57838 | **0.0037** | 0.2863 | 0.26253 | 0.3661 | 0.81955 | 0.4221 |
| Men can get HPV | 0.52698 | 0.14905 | 0.51626 | 0.57641 | 0.68818 | 0.30443 | **0.02735** |
| A person could have HPV for many years without knowing it | 0.70138 | 0.81325 | 0.31177 | 0.86828 | 0.73555 | 0.60076 | 0.61754 |
| The HPV vaccine is most effective if given to girls who have never had sex | 0.06729 | **0.00121** | 0.05531 | 0.44877 | 0.63318 | 0.99294 | **0.01743** |
| The HPV vaccine offers protection against cervical cancer | 0.71496 | 0.48898 | 0.64901 | 0.29347 | 1 | 1 | 0.26395 |
| Girls who have had the HPV vaccine do not need VIA when they are older | 0.63741 | 0.67034 | 0.55036 | 0.84894 | 0.82369 | 0.99804 | 0.76681 |
| Overall Score Category | 0.20603 | 0.10268 | 0.88106 | 0.11603 | 0.3581 | 0.45652 | 0.48152 |
