# Supplementary material for: Changes in attitudes toward the HPV vaccine in Kenya
Source: PLOS Glob Public Health. 2026 Oct 23;6(10):e0007508. doi: 10.1371/journal.pgph.0007508 (PMC13693870; doi:10.1371/journal.pgph.0007508)
Supplement: Supplementary file 5 [file pgph.0007508.s005.docx]

S4 Table: Baseline attitudes (2022) by demographic and by level of knowledge (about cervical cancer, HPV, and HPV vaccination)

| **Feature** | **Category** | **Category total** | **Attitude variable** | **Strongly agree** | **Somewhat agree** | **Somewhat disagree** | **Strongly disagree** | **Don't know** |
| --- | --- | --- | --- | --- | --- | --- | --- | --- |
| **Gender** | Female | 574 | HPV Vaccine is Important | 476 (82.9%) | 53 (9.2%) | 11 (1.9%) | 14 (2.4%) | 20 (3.5%) |
| **Gender** | Male | 519 | HPV Vaccine is Important | 441 (85.0%) | 45 (8.7%) | 5 (1.0%) | 15 (2.9%) | 13 (2.5%) |
| **Education Attainment** | Higher | 487 | HPV Vaccine is Important | 383 (78.6%) | 55 (11.3%) | 11 (2.3%) | 19 (3.9%) | 19 (3.9%) |
| **Education Attainment** | No school | 29 | HPV Vaccine is Important | 27 (93.1%) | 0 (0.0%) | 2 (6.9%) | 0 (0.0%) | 0 (0.0%) |
| **Education Attainment** | Primary | 235 | HPV Vaccine is Important | 213 (90.6%) | 12 (5.1%) | 1 (0.4%) | 4 (1.7%) | 5 (2.1%) |
| **Education Attainment** | Secondary | 342 | HPV Vaccine is Important | 294 (86.0%) | 31 (9.1%) | 2 (0.6%) | 6 (1.8%) | 9 (2.6%) |
| **Employment Status** | Formal Employment | 359 | HPV Vaccine is Important | 293 (81.6%) | 35 (9.7%) | 7 (1.9%) | 10 (2.8%) | 14 (3.9%) |
| **Employment Status** | Self-Employed/Informal | 614 | HPV Vaccine is Important | 523 (85.2%) | 53 (8.6%) | 9 (1.5%) | 16 (2.6%) | 13 (2.1%) |
| **Employment Status** | Unemployed | 117 | HPV Vaccine is Important | 98 (83.8%) | 10 (8.5%) | 0 (0.0%) | 3 (2.6%) | 6 (5.1%) |
| **Employment Status** | Missing | 3 | HPV Vaccine is Important | 3 (100.0%) | 0 (0.0%) | 0 (0.0%) | 0 (0.0%) | 0 (0.0%) |
| **Marital Status** | Partnered/Married | 857 | HPV Vaccine is Important | 729 (85.1%) | 75 (8.8%) | 10 (1.2%) | 21 (2.5%) | 22 (2.6%) |
| **Marital Status** | Previously Married | 84 | HPV Vaccine is Important | 66 (78.6%) | 9 (10.7%) | 1 (1.2%) | 2 (2.4%) | 6 (7.1%) |
| **Marital Status** | Single | 143 | HPV Vaccine is Important | 117 (81.8%) | 13 (9.1%) | 3 (2.1%) | 6 (4.2%) | 4 (2.8%) |
| **Marital Status** | Missing | 9 | HPV Vaccine is Important | 5 (55.6%) | 1 (11.1%) | 2 (22.2%) | 0 (0.0%) | 1 (11.1%) |
| **Household Income** | High | 138 | HPV Vaccine is Important | 112 (81.2%) | 13 (9.4%) | 2 (1.4%) | 5 (3.6%) | 6 (4.3%) |
| **Household Income** | Low | 678 | HPV Vaccine is Important | 582 (85.8%) | 55 (8.1%) | 8 (1.2%) | 13 (1.9%) | 20 (2.9%) |
| **Household Income** | Mid | 266 | HPV Vaccine is Important | 214 (80.5%) | 30 (11.3%) | 5 (1.9%) | 10 (3.8%) | 7 (2.6%) |
| **Household Income** | Missing | 11 | HPV Vaccine is Important | 9 (81.8%) | 0 (0.0%) | 1 (9.1%) | 1 (9.1%) | 0 (0.0%) |
| **Residence Area** | Rural | 467 | HPV Vaccine is Important | 409 (87.6%) | 33 (7.1%) | 5 (1.1%) | 10 (2.1%) | 10 (2.1%) |
| **Residence Area** | Town | 323 | HPV Vaccine is Important | 269 (83.3%) | 28 (8.7%) | 5 (1.5%) | 9 (2.8%) | 12 (3.7%) |
| **Residence Area** | Urban | 303 | HPV Vaccine is Important | 239 (78.9%) | 37 (12.2%) | 6 (2.0%) | 10 (3.3%) | 11 (3.6%) |
| **Religion** | Anglican | 115 | HPV Vaccine is Important | 97 (84.3%) | 13 (11.3%) | 1 (0.9%) | 2 (1.7%) | 2 (1.7%) |
| **Religion** | Catholic | 250 | HPV Vaccine is Important | 210 (84.0%) | 20 (8.0%) | 6 (2.4%) | 6 (2.4%) | 8 (3.2%) |
| **Religion** | Muslim | 58 | HPV Vaccine is Important | 48 (82.8%) | 5 (8.6%) | 0 (0.0%) | 3 (5.2%) | 2 (3.4%) |
| **Religion** | No Religion | 13 | HPV Vaccine is Important | 12 (92.3%) | 1 (7.7%) | 0 (0.0%) | 0 (0.0%) | 0 (0.0%) |
| **Religion** | Protestant/Pentecostal | 530 | HPV Vaccine is Important | 446 (84.2%) | 45 (8.5%) | 7 (1.3%) | 13 (2.5%) | 19 (3.6%) |
| **Religion** | SDA | 79 | HPV Vaccine is Important | 62 (78.5%) | 10 (12.7%) | 1 (1.3%) | 4 (5.1%) | 2 (2.5%) |
| **Religion** | Traditional/Tribal | 30 | HPV Vaccine is Important | 26 (86.7%) | 4 (13.3%) | 0 (0.0%) | 0 (0.0%) | 0 (0.0%) |
| **Religion** | Missing | 18 | HPV Vaccine is Important | 16 (88.9%) | 0 (0.0%) | 1 (5.6%) | 1 (5.6%) | 0 (0.0%) |
| **HPV Knowledge Score** | High | 295 | HPV Vaccine is Important | 266 (90.2%) | 21 (7.1%) | 2 (0.7%) | 4 (1.4%) | 2 (0.7%) |
| **HPV Knowledge Score** | Medium | 480 | HPV Vaccine is Important | 405 (84.4%) | 48 (10.0%) | 4 (0.8%) | 13 (2.7%) | 10 (2.1%) |
| **HPV Knowledge Score** | Low | 304 | HPV Vaccine is Important | 238 (78.3%) | 27 (8.9%) | 7 (2.3%) | 11 (3.6%) | 21 (6.9%) |
| **HPV Knowledge Score** | Missing | 14 | HPV Vaccine is Important | 8 (57.1%) | 2 (14.3%) | 3 (21.4%) | 1 (7.1%) | 0 (0.0%) |
| **Gender** | Female | 574 | HPV Vaccine is Effective | 477 (83.1%) | 58 (10.1%) | 9 (1.6%) | 15 (2.6%) | 15 (2.6%) |
| **Gender** | Male | 519 | HPV Vaccine is Effective | 448 (86.3%) | 41 (7.9%) | 7 (1.3%) | 10 (1.9%) | 13 (2.5%) |
| **Education Attainment** | Higher | 487 | HPV Vaccine is Effective | 387 (79.5%) | 60 (12.3%) | 9 (1.8%) | 14 (2.9%) | 17 (3.5%) |
| **Education Attainment** | No school | 29 | HPV Vaccine is Effective | 26 (89.7%) | 0 (0.0%) | 1 (3.4%) | 1 (3.4%) | 1 (3.4%) |
| **Education Attainment** | Primary | 235 | HPV Vaccine is Effective | 214 (91.1%) | 12 (5.1%) | 3 (1.3%) | 3 (1.3%) | 3 (1.3%) |
| **Education Attainment** | Secondary | 342 | HPV Vaccine is Effective | 298 (87.1%) | 27 (7.9%) | 3 (0.9%) | 7 (2.0%) | 7 (2.0%) |
| **Employment Status** | Formal Employment | 359 | HPV Vaccine is Effective | 292 (81.3%) | 43 (12.0%) | 4 (1.1%) | 8 (2.2%) | 12 (3.3%) |
| **Employment Status** | Self-Employed/Informal | 614 | HPV Vaccine is Effective | 531 (86.5%) | 46 (7.5%) | 10 (1.6%) | 14 (2.3%) | 13 (2.1%) |
| **Employment Status** | Unemployed | 117 | HPV Vaccine is Effective | 99 (84.6%) | 10 (8.5%) | 2 (1.7%) | 3 (2.6%) | 3 (2.6%) |
| **Employment Status** | Missing | 3 | HPV Vaccine is Effective | 3 (100.0%) | 0 (0.0%) | 0 (0.0%) | 0 (0.0%) | 0 (0.0%) |
| **Marital Status** | Partnered/Married | 857 | HPV Vaccine is Effective | 729 (85.1%) | 81 (9.5%) | 12 (1.4%) | 16 (1.9%) | 19 (2.2%) |
| **Marital Status** | Previously Married | 84 | HPV Vaccine is Effective | 69 (82.1%) | 6 (7.1%) | 0 (0.0%) | 3 (3.6%) | 6 (7.1%) |
| **Marital Status** | Single | 143 | HPV Vaccine is Effective | 120 (83.9%) | 12 (8.4%) | 3 (2.1%) | 6 (4.2%) | 2 (1.4%) |
| **Marital Status** | Missing | 9 | HPV Vaccine is Effective | 7 (77.8%) | 0 (0.0%) | 1 (11.1%) | 0 (0.0%) | 1 (11.1%) |
| **Household Income** | High | 138 | HPV Vaccine is Effective | 117 (84.8%) | 13 (9.4%) | 1 (0.7%) | 1 (0.7%) | 6 (4.3%) |
| **Household Income** | Low | 678 | HPV Vaccine is Effective | 584 (86.1%) | 53 (7.8%) | 8 (1.2%) | 16 (2.4%) | 17 (2.5%) |
| **Household Income** | Mid | 266 | HPV Vaccine is Effective | 216 (81.2%) | 33 (12.4%) | 6 (2.3%) | 6 (2.3%) | 5 (1.9%) |
| **Household Income** | Missing | 11 | HPV Vaccine is Effective | 8 (72.7%) | 0 (0.0%) | 1 (9.1%) | 2 (18.2%) | 0 (0.0%) |
| **Residence Area** | Rural | 467 | HPV Vaccine is Effective | 408 (87.4%) | 36 (7.7%) | 4 (0.9%) | 11 (2.4%) | 8 (1.7%) |
| **Residence Area** | Town | 323 | HPV Vaccine is Effective | 270 (83.6%) | 31 (9.6%) | 5 (1.5%) | 7 (2.2%) | 10 (3.1%) |
| **Residence Area** | Urban | 303 | HPV Vaccine is Effective | 247 (81.5%) | 32 (10.6%) | 7 (2.3%) | 7 (2.3%) | 10 (3.3%) |
| **Religion** | Anglican | 115 | HPV Vaccine is Effective | 103 (89.6%) | 6 (5.2%) | 1 (0.9%) | 3 (2.6%) | 2 (1.7%) |
| **Religion** | Catholic | 250 | HPV Vaccine is Effective | 207 (82.8%) | 26 (10.4%) | 5 (2.0%) | 5 (2.0%) | 7 (2.8%) |
| **Religion** | Muslim | 58 | HPV Vaccine is Effective | 49 (84.5%) | 5 (8.6%) | 1 (1.7%) | 2 (3.4%) | 1 (1.7%) |
| **Religion** | No Religion | 13 | HPV Vaccine is Effective | 11 (84.6%) | 2 (15.4%) | 0 (0.0%) | 0 (0.0%) | 0 (0.0%) |
| **Religion** | Protestant/Pentecostal | 530 | HPV Vaccine is Effective | 448 (84.5%) | 47 (8.9%) | 8 (1.5%) | 11 (2.1%) | 16 (3.0%) |
| **Religion** | SDA | 79 | HPV Vaccine is Effective | 63 (79.7%) | 10 (12.7%) | 1 (1.3%) | 3 (3.8%) | 2 (2.5%) |
| **Religion** | Traditional/Tribal | 30 | HPV Vaccine is Effective | 27 (90.0%) | 3 (10.0%) | 0 (0.0%) | 0 (0.0%) | 0 (0.0%) |
| **Religion** | Missing | 18 | HPV Vaccine is Effective | 17 (94.4%) | 0 (0.0%) | 0 (0.0%) | 1 (5.6%) | 0 (0.0%) |
| **HPV Knowledge Score** | High | 295 | HPV Vaccine is Effective | 268 (90.8%) | 20 (6.8%) | 2 (0.7%) | 3 (1.0%) | 2 (0.7%) |
| **HPV Knowledge Score** | Medium | 480 | HPV Vaccine is Effective | 405 (84.4%) | 50 (10.4%) | 3 (0.6%) | 12 (2.5%) | 10 (2.1%) |
| **HPV Knowledge Score** | Low | 304 | HPV Vaccine is Effective | 242 (79.6%) | 27 (8.9%) | 10 (3.3%) | 9 (3.0%) | 16 (5.3%) |
| **HPV Knowledge Score** | Missing | 14 | HPV Vaccine is Effective | 10 (71.4%) | 2 (14.3%) | 1 (7.1%) | 1 (7.1%) | 0 (0.0%) |
| **Gender** | Female | 574 | Clinician Guidance | 501 (87.3%) | 52 (9.1%) | 7 (1.2%) | 9 (1.6%) | 5 (0.9%) |
| **Gender** | Male | 519 | Clinician Guidance | 468 (90.2%) | 30 (5.8%) | 7 (1.3%) | 12 (2.3%) | 2 (0.4%) |
| **Education Attainment** | Higher | 487 | Clinician Guidance | 419 (86.0%) | 42 (8.6%) | 10 (2.1%) | 12 (2.5%) | 4 (0.8%) |
| **Education Attainment** | No school | 29 | Clinician Guidance | 27 (93.1%) | 2 (6.9%) | 0 (0.0%) | 0 (0.0%) | 0 (0.0%) |
| **Education Attainment** | Primary | 235 | Clinician Guidance | 216 (91.9%) | 14 (6.0%) | 2 (0.9%) | 1 (0.4%) | 2 (0.9%) |
| **Education Attainment** | Secondary | 342 | Clinician Guidance | 307 (89.8%) | 24 (7.0%) | 2 (0.6%) | 8 (2.3%) | 1 (0.3%) |
| **Employment Status** | Formal Employment | 359 | Clinician Guidance | 310 (86.4%) | 30 (8.4%) | 8 (2.2%) | 8 (2.2%) | 3 (0.8%) |
| **Employment Status** | Self-Employed/Informal | 614 | Clinician Guidance | 558 (90.9%) | 35 (5.7%) | 5 (0.8%) | 13 (2.1%) | 3 (0.5%) |
| **Employment Status** | Unemployed | 117 | Clinician Guidance | 98 (83.8%) | 17 (14.5%) | 1 (0.9%) | 0 (0.0%) | 1 (0.9%) |
| **Employment Status** | Missing | 3 | Clinician Guidance | 3 (100.0%) | 0 (0.0%) | 0 (0.0%) | 0 (0.0%) | 0 (0.0%) |
| **Marital Status** | Partnered/Married | 857 | Clinician Guidance | 761 (88.8%) | 63 (7.4%) | 13 (1.5%) | 16 (1.9%) | 4 (0.5%) |
| **Marital Status** | Previously Married | 84 | Clinician Guidance | 76 (90.5%) | 7 (8.3%) | 0 (0.0%) | 1 (1.2%) | 0 (0.0%) |
| **Marital Status** | Single | 143 | Clinician Guidance | 124 (86.7%) | 12 (8.4%) | 1 (0.7%) | 3 (2.1%) | 3 (2.1%) |
| **Marital Status** | Missing | 9 | Clinician Guidance | 8 (88.9%) | 0 (0.0%) | 0 (0.0%) | 1 (11.1%) | 0 (0.0%) |
| **Household Income** | High | 138 | Clinician Guidance | 115 (83.3%) | 15 (10.9%) | 3 (2.2%) | 4 (2.9%) | 1 (0.7%) |
| **Household Income** | Low | 678 | Clinician Guidance | 607 (89.5%) | 49 (7.2%) | 5 (0.7%) | 12 (1.8%) | 5 (0.7%) |
| **Household Income** | Mid | 266 | Clinician Guidance | 237 (89.1%) | 18 (6.8%) | 6 (2.3%) | 4 (1.5%) | 1 (0.4%) |
| **Household Income** | Missing | 11 | Clinician Guidance | 10 (90.9%) | 0 (0.0%) | 0 (0.0%) | 1 (9.1%) | 0 (0.0%) |
| **Residence Area** | Rural | 467 | Clinician Guidance | 432 (92.5%) | 26 (5.6%) | 3 (0.6%) | 5 (1.1%) | 1 (0.2%) |
| **Residence Area** | Town | 323 | Clinician Guidance | 276 (85.4%) | 25 (7.7%) | 6 (1.9%) | 11 (3.4%) | 5 (1.5%) |
| **Residence Area** | Urban | 303 | Clinician Guidance | 261 (86.1%) | 31 (10.2%) | 5 (1.7%) | 5 (1.7%) | 1 (0.3%) |
| **Religion** | Anglican | 115 | Clinician Guidance | 102 (88.7%) | 9 (7.8%) | 1 (0.9%) | 3 (2.6%) | 0 (0.0%) |
| **Religion** | Catholic | 250 | Clinician Guidance | 219 (87.6%) | 20 (8.0%) | 3 (1.2%) | 6 (2.4%) | 2 (0.8%) |
| **Religion** | Muslim | 58 | Clinician Guidance | 54 (93.1%) | 3 (5.2%) | 0 (0.0%) | 1 (1.7%) | 0 (0.0%) |
| **Religion** | No Religion | 13 | Clinician Guidance | 10 (76.9%) | 2 (15.4%) | 1 (7.7%) | 0 (0.0%) | 0 (0.0%) |
| **Religion** | Protestant/Pentecostal | 530 | Clinician Guidance | 472 (89.1%) | 38 (7.2%) | 7 (1.3%) | 8 (1.5%) | 5 (0.9%) |
| **Religion** | SDA | 79 | Clinician Guidance | 71 (89.9%) | 4 (5.1%) | 2 (2.5%) | 2 (2.5%) | 0 (0.0%) |
| **Religion** | Traditional/Tribal | 30 | Clinician Guidance | 25 (83.3%) | 5 (16.7%) | 0 (0.0%) | 0 (0.0%) | 0 (0.0%) |
| **Religion** | Missing | 18 | Clinician Guidance | 16 (88.9%) | 1 (5.6%) | 0 (0.0%) | 1 (5.6%) | 0 (0.0%) |
| **HPV Knowledge Score** | High | 295 | Clinician Guidance | 268 (90.8%) | 19 (6.4%) | 3 (1.0%) | 4 (1.4%) | 1 (0.3%) |
| **HPV Knowledge Score** | Medium | 480 | Clinician Guidance | 426 (88.8%) | 39 (8.1%) | 4 (0.8%) | 8 (1.7%) | 3 (0.6%) |
| **HPV Knowledge Score** | Low | 304 | Clinician Guidance | 266 (87.5%) | 22 (7.2%) | 4 (1.3%) | 9 (3.0%) | 3 (1.0%) |
| **HPV Knowledge Score** | Missing | 14 | Clinician Guidance | 9 (64.3%) | 2 (14.3%) | 3 (21.4%) | 0 (0.0%) | 0 (0.0%) |
| **Gender** | Female | 574 | Moderate Side Effects | 184 (32.1%) | 103 (17.9%) | 47 (8.2%) | 210 (36.6%) | 30 (5.2%) |
| **Gender** | Male | 519 | Moderate Side Effects | 160 (30.8%) | 69 (13.3%) | 38 (7.3%) | 219 (42.2%) | 33 (6.4%) |
| **Education Attainment** | Higher | 487 | Moderate Side Effects | 180 (37.0%) | 84 (17.2%) | 31 (6.4%) | 156 (32.0%) | 36 (7.4%) |
| **Education Attainment** | No school | 29 | Moderate Side Effects | 8 (27.6%) | 3 (10.3%) | 5 (17.2%) | 11 (37.9%) | 2 (6.9%) |
| **Education Attainment** | Primary | 235 | Moderate Side Effects | 64 (27.2%) | 29 (12.3%) | 25 (10.6%) | 107 (45.5%) | 10 (4.3%) |
| **Education Attainment** | Secondary | 342 | Moderate Side Effects | 92 (26.9%) | 56 (16.4%) | 24 (7.0%) | 155 (45.3%) | 15 (4.4%) |
| **Employment Status** | Formal Employment | 359 | Moderate Side Effects | 129 (35.9%) | 55 (15.3%) | 23 (6.4%) | 123 (34.3%) | 29 (8.1%) |
| **Employment Status** | Self-Employed/Informal | 614 | Moderate Side Effects | 176 (28.7%) | 93 (15.1%) | 51 (8.3%) | 264 (43.0%) | 30 (4.9%) |
| **Employment Status** | Unemployed | 117 | Moderate Side Effects | 39 (33.3%) | 23 (19.7%) | 11 (9.4%) | 40 (34.2%) | 4 (3.4%) |
| **Employment Status** | Missing | 3 | Moderate Side Effects | 0 (0.0%) | 1 (33.3%) | 0 (0.0%) | 2 (66.7%) | 0 (0.0%) |
| **Marital Status** | Partnered/Married | 857 | Moderate Side Effects | 255 (29.8%) | 137 (16.0%) | 61 (7.1%) | 358 (41.8%) | 46 (5.4%) |
| **Marital Status** | Previously Married | 84 | Moderate Side Effects | 32 (38.1%) | 11 (13.1%) | 5 (6.0%) | 28 (33.3%) | 8 (9.5%) |
| **Marital Status** | Single | 143 | Moderate Side Effects | 52 (36.4%) | 23 (16.1%) | 19 (13.3%) | 42 (29.4%) | 7 (4.9%) |
| **Marital Status** | Missing | 9 | Moderate Side Effects | 5 (55.6%) | 1 (11.1%) | 0 (0.0%) | 1 (11.1%) | 2 (22.2%) |
| **Household Income** | High | 138 | Moderate Side Effects | 43 (31.2%) | 27 (19.6%) | 6 (4.3%) | 56 (40.6%) | 6 (4.3%) |
| **Household Income** | Low | 678 | Moderate Side Effects | 220 (32.4%) | 108 (15.9%) | 55 (8.1%) | 254 (37.5%) | 41 (6.0%) |
| **Household Income** | Mid | 266 | Moderate Side Effects | 78 (29.3%) | 36 (13.5%) | 23 (8.6%) | 113 (42.5%) | 16 (6.0%) |
| **Household Income** | Missing | 11 | Moderate Side Effects | 3 (27.3%) | 1 (9.1%) | 1 (9.1%) | 6 (54.5%) | 0 (0.0%) |
| **Residence Area** | Rural | 467 | Moderate Side Effects | 135 (28.9%) | 65 (13.9%) | 34 (7.3%) | 210 (45.0%) | 23 (4.9%) |
| **Residence Area** | Town | 323 | Moderate Side Effects | 109 (33.7%) | 50 (15.5%) | 25 (7.7%) | 123 (38.1%) | 16 (5.0%) |
| **Residence Area** | Urban | 303 | Moderate Side Effects | 100 (33.0%) | 57 (18.8%) | 26 (8.6%) | 96 (31.7%) | 24 (7.9%) |
| **Religion** | Anglican | 115 | Moderate Side Effects | 34 (29.6%) | 22 (19.1%) | 5 (4.3%) | 49 (42.6%) | 5 (4.3%) |
| **Religion** | Catholic | 250 | Moderate Side Effects | 72 (28.8%) | 45 (18.0%) | 29 (11.6%) | 92 (36.8%) | 12 (4.8%) |
| **Religion** | Muslim | 58 | Moderate Side Effects | 22 (37.9%) | 9 (15.5%) | 2 (3.4%) | 21 (36.2%) | 4 (6.9%) |
| **Religion** | No Religion | 13 | Moderate Side Effects | 3 (23.1%) | 1 (7.7%) | 2 (15.4%) | 7 (53.8%) | 0 (0.0%) |
| **Religion** | Protestant/Pentecostal | 530 | Moderate Side Effects | 169 (31.9%) | 72 (13.6%) | 35 (6.6%) | 215 (40.6%) | 39 (7.4%) |
| **Religion** | SDA | 79 | Moderate Side Effects | 27 (34.2%) | 15 (19.0%) | 10 (12.7%) | 25 (31.6%) | 2 (2.5%) |
| **Religion** | Traditional/Tribal | 30 | Moderate Side Effects | 8 (26.7%) | 6 (20.0%) | 2 (6.7%) | 14 (46.7%) | 0 (0.0%) |
| **Religion** | Missing | 18 | Moderate Side Effects | 9 (50.0%) | 2 (11.1%) | 0 (0.0%) | 6 (33.3%) | 1 (5.6%) |
| **HPV Knowledge Score** | High | 295 | Moderate Side Effects | 93 (31.5%) | 38 (12.9%) | 20 (6.8%) | 136 (46.1%) | 8 (2.7%) |
| **HPV Knowledge Score** | Medium | 480 | Moderate Side Effects | 142 (29.6%) | 90 (18.8%) | 29 (6.0%) | 196 (40.8%) | 23 (4.8%) |
| **HPV Knowledge Score** | Low | 304 | Moderate Side Effects | 105 (34.5%) | 41 (13.5%) | 34 (11.2%) | 94 (30.9%) | 30 (9.9%) |
| **HPV Knowledge Score** | Missing | 14 | Moderate Side Effects | 4 (28.6%) | 3 (21.4%) | 2 (14.3%) | 3 (21.4%) | 2 (14.3%) |
| **Gender** | Female | 574 | Serious Side Effects | 169 (29.4%) | 98 (17.1%) | 53 (9.2%) | 222 (38.7%) | 32 (5.6%) |
| **Gender** | Male | 519 | Serious Side Effects | 148 (28.5%) | 69 (13.3%) | 36 (6.9%) | 231 (44.5%) | 35 (6.7%) |
| **Education Attainment** | Higher | 487 | Serious Side Effects | 169 (34.7%) | 72 (14.8%) | 37 (7.6%) | 168 (34.5%) | 41 (8.4%) |
| **Education Attainment** | No school | 29 | Serious Side Effects | 6 (20.7%) | 4 (13.8%) | 6 (20.7%) | 11 (37.9%) | 2 (6.9%) |
| **Education Attainment** | Primary | 235 | Serious Side Effects | 58 (24.7%) | 30 (12.8%) | 16 (6.8%) | 119 (50.6%) | 12 (5.1%) |
| **Education Attainment** | Secondary | 342 | Serious Side Effects | 84 (24.6%) | 61 (17.8%) | 30 (8.8%) | 155 (45.3%) | 12 (3.5%) |
| **Employment Status** | Formal Employment | 359 | Serious Side Effects | 113 (31.5%) | 51 (14.2%) | 31 (8.6%) | 133 (37.0%) | 31 (8.6%) |
| **Employment Status** | Self-Employed/Informal | 614 | Serious Side Effects | 163 (26.5%) | 97 (15.8%) | 49 (8.0%) | 275 (44.8%) | 30 (4.9%) |
| **Employment Status** | Unemployed | 117 | Serious Side Effects | 41 (35.0%) | 19 (16.2%) | 9 (7.7%) | 42 (35.9%) | 6 (5.1%) |
| **Employment Status** | Missing | 3 | Serious Side Effects | 0 (0.0%) | 0 (0.0%) | 0 (0.0%) | 3 (100.0%) | 0 (0.0%) |
| **Marital Status** | Partnered/Married | 857 | Serious Side Effects | 241 (28.1%) | 128 (14.9%) | 64 (7.5%) | 375 (43.8%) | 49 (5.7%) |
| **Marital Status** | Previously Married | 84 | Serious Side Effects | 26 (31.0%) | 13 (15.5%) | 4 (4.8%) | 33 (39.3%) | 8 (9.5%) |
| **Marital Status** | Single | 143 | Serious Side Effects | 45 (31.5%) | 25 (17.5%) | 20 (14.0%) | 45 (31.5%) | 8 (5.6%) |
| **Marital Status** | Missing | 9 | Serious Side Effects | 5 (55.6%) | 1 (11.1%) | 1 (11.1%) | 0 (0.0%) | 2 (22.2%) |
| **Household Income** | High | 138 | Serious Side Effects | 43 (31.2%) | 25 (18.1%) | 11 (8.0%) | 51 (37.0%) | 8 (5.8%) |
| **Household Income** | Low | 678 | Serious Side Effects | 203 (29.9%) | 106 (15.6%) | 57 (8.4%) | 271 (40.0%) | 41 (6.0%) |
| **Household Income** | Mid | 266 | Serious Side Effects | 68 (25.6%) | 35 (13.2%) | 20 (7.5%) | 126 (47.4%) | 17 (6.4%) |
| **Household Income** | Missing | 11 | Serious Side Effects | 3 (27.3%) | 1 (9.1%) | 1 (9.1%) | 5 (45.5%) | 1 (9.1%) |
| **Residence Area** | Rural | 467 | Serious Side Effects | 128 (27.4%) | 68 (14.6%) | 32 (6.9%) | 215 (46.0%) | 24 (5.1%) |
| **Residence Area** | Town | 323 | Serious Side Effects | 97 (30.0%) | 49 (15.2%) | 24 (7.4%) | 137 (42.4%) | 16 (5.0%) |
| **Residence Area** | Urban | 303 | Serious Side Effects | 92 (30.4%) | 50 (16.5%) | 33 (10.9%) | 101 (33.3%) | 27 (8.9%) |
| **Religion** | Anglican | 115 | Serious Side Effects | 30 (26.1%) | 24 (20.9%) | 6 (5.2%) | 49 (42.6%) | 6 (5.2%) |
| **Religion** | Catholic | 250 | Serious Side Effects | 77 (30.8%) | 44 (17.6%) | 29 (11.6%) | 89 (35.6%) | 11 (4.4%) |
| **Religion** | Muslim | 58 | Serious Side Effects | 20 (34.5%) | 11 (19.0%) | 2 (3.4%) | 21 (36.2%) | 4 (6.9%) |
| **Religion** | No Religion | 13 | Serious Side Effects | 2 (15.4%) | 1 (7.7%) | 2 (15.4%) | 7 (53.8%) | 1 (7.7%) |
| **Religion** | Protestant/Pentecostal | 530 | Serious Side Effects | 148 (27.9%) | 66 (12.5%) | 36 (6.8%) | 240 (45.3%) | 40 (7.5%) |
| **Religion** | SDA | 79 | Serious Side Effects | 27 (34.2%) | 13 (16.5%) | 10 (12.7%) | 27 (34.2%) | 2 (2.5%) |
| **Religion** | Traditional/Tribal | 30 | Serious Side Effects | 6 (20.0%) | 6 (20.0%) | 4 (13.3%) | 13 (43.3%) | 1 (3.3%) |
| **Religion** | Missing | 18 | Serious Side Effects | 7 (38.9%) | 2 (11.1%) | 0 (0.0%) | 7 (38.9%) | 2 (11.1%) |
| **HPV Knowledge Score** | High | 295 | Serious Side Effects | 85 (28.8%) | 38 (12.9%) | 20 (6.8%) | 142 (48.1%) | 10 (3.4%) |
| **HPV Knowledge Score** | Medium | 480 | Serious Side Effects | 135 (28.1%) | 86 (17.9%) | 34 (7.1%) | 203 (42.3%) | 22 (4.6%) |
| **HPV Knowledge Score** | Low | 304 | Serious Side Effects | 93 (30.6%) | 40 (13.2%) | 32 (10.5%) | 106 (34.9%) | 33 (10.9%) |
| **HPV Knowledge Score** | Missing | 14 | Serious Side Effects | 4 (28.6%) | 3 (21.4%) | 3 (21.4%) | 2 (14.3%) | 2 (14.3%) |
| **Gender** | Female | 574 | Long-term Side Effects | 183 (31.9%) | 85 (14.8%) | 45 (7.8%) | 229 (39.9%) | 32 (5.6%) |
| **Gender** | Male | 519 | Long-term Side Effects | 164 (31.6%) | 68 (13.1%) | 35 (6.7%) | 215 (41.4%) | 37 (7.1%) |
| **Education Attainment** | Higher | 487 | Long-term Side Effects | 179 (36.8%) | 67 (13.8%) | 35 (7.2%) | 168 (34.5%) | 38 (7.8%) |
| **Education Attainment** | No school | 29 | Long-term Side Effects | 5 (17.2%) | 4 (13.8%) | 5 (17.2%) | 13 (44.8%) | 2 (6.9%) |
| **Education Attainment** | Primary | 235 | Long-term Side Effects | 65 (27.7%) | 29 (12.3%) | 15 (6.4%) | 114 (48.5%) | 12 (5.1%) |
| **Education Attainment** | Secondary | 342 | Long-term Side Effects | 98 (28.7%) | 53 (15.5%) | 25 (7.3%) | 149 (43.6%) | 17 (5.0%) |
| **Employment Status** | Formal Employment | 359 | Long-term Side Effects | 124 (34.5%) | 45 (12.5%) | 31 (8.6%) | 129 (35.9%) | 30 (8.4%) |
| **Employment Status** | Self-Employed/Informal | 614 | Long-term Side Effects | 186 (30.3%) | 90 (14.7%) | 38 (6.2%) | 267 (43.5%) | 33 (5.4%) |
| **Employment Status** | Unemployed | 117 | Long-term Side Effects | 37 (31.6%) | 18 (15.4%) | 11 (9.4%) | 45 (38.5%) | 6 (5.1%) |
| **Employment Status** | Missing | 3 | Long-term Side Effects | 0 (0.0%) | 0 (0.0%) | 0 (0.0%) | 3 (100.0%) | 0 (0.0%) |
| **Marital Status** | Partnered/Married | 857 | Long-term Side Effects | 269 (31.4%) | 116 (13.5%) | 64 (7.5%) | 356 (41.5%) | 52 (6.1%) |
| **Marital Status** | Previously Married | 84 | Long-term Side Effects | 27 (32.1%) | 13 (15.5%) | 4 (4.8%) | 32 (38.1%) | 8 (9.5%) |
| **Marital Status** | Single | 143 | Long-term Side Effects | 45 (31.5%) | 23 (16.1%) | 11 (7.7%) | 56 (39.2%) | 8 (5.6%) |
| **Marital Status** | Missing | 9 | Long-term Side Effects | 6 (66.7%) | 1 (11.1%) | 1 (11.1%) | 0 (0.0%) | 1 (11.1%) |
| **Household Income** | High | 138 | Long-term Side Effects | 47 (34.1%) | 24 (17.4%) | 9 (6.5%) | 49 (35.5%) | 9 (6.5%) |
| **Household Income** | Low | 678 | Long-term Side Effects | 221 (32.6%) | 92 (13.6%) | 50 (7.4%) | 272 (40.1%) | 43 (6.3%) |
| **Household Income** | Mid | 266 | Long-term Side Effects | 75 (28.2%) | 37 (13.9%) | 20 (7.5%) | 118 (44.4%) | 16 (6.0%) |
| **Household Income** | Missing | 11 | Long-term Side Effects | 4 (36.4%) | 0 (0.0%) | 1 (9.1%) | 5 (45.5%) | 1 (9.1%) |
| **Residence Area** | Rural | 467 | Long-term Side Effects | 126 (27.0%) | 71 (15.2%) | 28 (6.0%) | 218 (46.7%) | 24 (5.1%) |
| **Residence Area** | Town | 323 | Long-term Side Effects | 109 (33.7%) | 40 (12.4%) | 26 (8.0%) | 131 (40.6%) | 17 (5.3%) |
| **Residence Area** | Urban | 303 | Long-term Side Effects | 112 (37.0%) | 42 (13.9%) | 26 (8.6%) | 95 (31.4%) | 28 (9.2%) |
| **Religion** | Anglican | 115 | Long-term Side Effects | 40 (34.8%) | 21 (18.3%) | 4 (3.5%) | 43 (37.4%) | 7 (6.1%) |
| **Religion** | Catholic | 250 | Long-term Side Effects | 81 (32.4%) | 45 (18.0%) | 24 (9.6%) | 88 (35.2%) | 12 (4.8%) |
| **Religion** | Muslim | 58 | Long-term Side Effects | 20 (34.5%) | 10 (17.2%) | 2 (3.4%) | 23 (39.7%) | 3 (5.2%) |
| **Religion** | No Religion | 13 | Long-term Side Effects | 2 (15.4%) | 1 (7.7%) | 2 (15.4%) | 8 (61.5%) | 0 (0.0%) |
| **Religion** | Protestant/Pentecostal | 530 | Long-term Side Effects | 164 (30.9%) | 54 (10.2%) | 36 (6.8%) | 234 (44.2%) | 42 (7.9%) |
| **Religion** | SDA | 79 | Long-term Side Effects | 27 (34.2%) | 11 (13.9%) | 8 (10.1%) | 31 (39.2%) | 2 (2.5%) |
| **Religion** | Traditional/Tribal | 30 | Long-term Side Effects | 5 (16.7%) | 8 (26.7%) | 4 (13.3%) | 12 (40.0%) | 1 (3.3%) |
| **Religion** | Missing | 18 | Long-term Side Effects | 8 (44.4%) | 3 (16.7%) | 0 (0.0%) | 5 (27.8%) | 2 (11.1%) |
| **HPV Knowledge Score** | High | 295 | Long-term Side Effects | 104 (35.3%) | 29 (9.8%) | 18 (6.1%) | 134 (45.4%) | 10 (3.4%) |
| **HPV Knowledge Score** | Medium | 480 | Long-term Side Effects | 143 (29.8%) | 80 (16.7%) | 36 (7.5%) | 200 (41.7%) | 21 (4.4%) |
| **HPV Knowledge Score** | Low | 304 | Long-term Side Effects | 95 (31.2%) | 42 (13.8%) | 26 (8.6%) | 107 (35.2%) | 34 (11.2%) |
| **HPV Knowledge Score** | Missing | 14 | Long-term Side Effects | 5 (35.7%) | 2 (14.3%) | 0 (0.0%) | 3 (21.4%) | 4 (28.6%) |
| **Gender** | Female | 574 | HPV Vaccine is Risky | 78 (13.6%) | 55 (9.6%) | 57 (9.9%) | 277 (48.3%) | 107 (18.6%) |
| **Gender** | Male | 519 | HPV Vaccine is Risky | 74 (14.3%) | 33 (6.4%) | 32 (6.2%) | 276 (53.2%) | 104 (20.0%) |
| **Education Attainment** | Higher | 487 | HPV Vaccine is Risky | 60 (12.3%) | 46 (9.4%) | 40 (8.2%) | 243 (49.9%) | 98 (20.1%) |
| **Education Attainment** | No school | 29 | HPV Vaccine is Risky | 6 (20.7%) | 0 (0.0%) | 2 (6.9%) | 14 (48.3%) | 7 (24.1%) |
| **Education Attainment** | Primary | 235 | HPV Vaccine is Risky | 48 (20.4%) | 19 (8.1%) | 15 (6.4%) | 114 (48.5%) | 39 (16.6%) |
| **Education Attainment** | Secondary | 342 | HPV Vaccine is Risky | 38 (11.1%) | 23 (6.7%) | 32 (9.4%) | 182 (53.2%) | 67 (19.6%) |
| **Employment Status** | Formal Employment | 359 | HPV Vaccine is Risky | 39 (10.9%) | 25 (7.0%) | 34 (9.5%) | 185 (51.5%) | 76 (21.2%) |
| **Employment Status** | Self-Employed/Informal | 614 | HPV Vaccine is Risky | 88 (14.3%) | 48 (7.8%) | 41 (6.7%) | 319 (52.0%) | 118 (19.2%) |
| **Employment Status** | Unemployed | 117 | HPV Vaccine is Risky | 25 (21.4%) | 14 (12.0%) | 14 (12.0%) | 47 (40.2%) | 17 (14.5%) |
| **Employment Status** | Missing | 3 | HPV Vaccine is Risky | 0 (0.0%) | 1 (33.3%) | 0 (0.0%) | 2 (66.7%) | 0 (0.0%) |
| **Marital Status** | Partnered/Married | 857 | HPV Vaccine is Risky | 112 (13.1%) | 70 (8.2%) | 66 (7.7%) | 443 (51.7%) | 166 (19.4%) |
| **Marital Status** | Previously Married | 84 | HPV Vaccine is Risky | 14 (16.7%) | 6 (7.1%) | 8 (9.5%) | 38 (45.2%) | 18 (21.4%) |
| **Marital Status** | Single | 143 | HPV Vaccine is Risky | 24 (16.8%) | 12 (8.4%) | 14 (9.8%) | 69 (48.3%) | 24 (16.8%) |
| **Marital Status** | Missing | 9 | HPV Vaccine is Risky | 2 (22.2%) | 0 (0.0%) | 1 (11.1%) | 3 (33.3%) | 3 (33.3%) |
| **Household Income** | High | 138 | HPV Vaccine is Risky | 15 (10.9%) | 14 (10.1%) | 10 (7.2%) | 76 (55.1%) | 23 (16.7%) |
| **Household Income** | Low | 678 | HPV Vaccine is Risky | 106 (15.6%) | 57 (8.4%) | 52 (7.7%) | 334 (49.3%) | 129 (19.0%) |
| **Household Income** | Mid | 266 | HPV Vaccine is Risky | 27 (10.2%) | 17 (6.4%) | 27 (10.2%) | 137 (51.5%) | 58 (21.8%) |
| **Household Income** | Missing | 11 | HPV Vaccine is Risky | 4 (36.4%) | 0 (0.0%) | 0 (0.0%) | 6 (54.5%) | 1 (9.1%) |
| **Residence Area** | Rural | 467 | HPV Vaccine is Risky | 66 (14.1%) | 37 (7.9%) | 37 (7.9%) | 254 (54.4%) | 73 (15.6%) |
| **Residence Area** | Town | 323 | HPV Vaccine is Risky | 43 (13.3%) | 26 (8.0%) | 23 (7.1%) | 165 (51.1%) | 66 (20.4%) |
| **Residence Area** | Urban | 303 | HPV Vaccine is Risky | 43 (14.2%) | 25 (8.3%) | 29 (9.6%) | 134 (44.2%) | 72 (23.8%) |
| **Religion** | Anglican | 115 | HPV Vaccine is Risky | 15 (13.0%) | 7 (6.1%) | 12 (10.4%) | 58 (50.4%) | 23 (20.0%) |
| **Religion** | Catholic | 250 | HPV Vaccine is Risky | 31 (12.4%) | 22 (8.8%) | 28 (11.2%) | 124 (49.6%) | 45 (18.0%) |
| **Religion** | Muslim | 58 | HPV Vaccine is Risky | 6 (10.3%) | 7 (12.1%) | 2 (3.4%) | 30 (51.7%) | 13 (22.4%) |
| **Religion** | No Religion | 13 | HPV Vaccine is Risky | 1 (7.7%) | 1 (7.7%) | 2 (15.4%) | 6 (46.2%) | 3 (23.1%) |
| **Religion** | Protestant/Pentecostal | 530 | HPV Vaccine is Risky | 81 (15.3%) | 40 (7.5%) | 24 (4.5%) | 275 (51.9%) | 110 (20.8%) |
| **Religion** | SDA | 79 | HPV Vaccine is Risky | 9 (11.4%) | 11 (13.9%) | 13 (16.5%) | 35 (44.3%) | 11 (13.9%) |
| **Religion** | Traditional/Tribal | 30 | HPV Vaccine is Risky | 6 (20.0%) | 0 (0.0%) | 5 (16.7%) | 16 (53.3%) | 3 (10.0%) |
| **Religion** | Missing | 18 | HPV Vaccine is Risky | 3 (16.7%) | 0 (0.0%) | 3 (16.7%) | 9 (50.0%) | 3 (16.7%) |
| **HPV Knowledge Score** | High | 295 | HPV Vaccine is Risky | 55 (18.6%) | 21 (7.1%) | 18 (6.1%) | 169 (57.3%) | 32 (10.8%) |
| **HPV Knowledge Score** | Medium | 480 | HPV Vaccine is Risky | 56 (11.7%) | 44 (9.2%) | 43 (9.0%) | 244 (50.8%) | 93 (19.4%) |
| **HPV Knowledge Score** | Low | 304 | HPV Vaccine is Risky | 38 (12.5%) | 19 (6.2%) | 27 (8.9%) | 137 (45.1%) | 83 (27.3%) |
| **HPV Knowledge Score** | Missing | 14 | HPV Vaccine is Risky | 3 (21.4%) | 4 (28.6%) | 1 (7.1%) | 3 (21.4%) | 3 (21.4%) |
