# Supplementary material for: Changes in attitudes toward the HPV vaccine in Kenya
Source: PLOS Glob Public Health. 2026 Oct 23;6(10):e0007508. doi: 10.1371/journal.pgph.0007508 (PMC13693870; doi:10.1371/journal.pgph.0007508)
Supplement: Supplementary file 6 [file pgph.0007508.s006.docx]

S6 Table: Attitude changes (2022 to 2024) by each question about knowledge

| **Feature** | **Category** | **Category total** | **Attitude variable** | **Positive** | **No Change** | **Negative** | **Other** |
| --- | --- | --- | --- | --- | --- | --- | --- |
| **HPV can cause cervical cancer** | Yes | 703 | HPV Vaccine is Important | 73 (10.4%) | 541 (77.0%) | 64 (9.1%) | 25 (3.6%) |
| **HPV can cause cervical cancer** | No | 390 | HPV Vaccine is Important | 41 (10.5%) | 290 (74.4%) | 33 (8.5%) | 26 (6.7%) |
| **HPV can be passed on during sexual intercourse** | Yes | 605 | HPV Vaccine is Important | 65 (10.7%) | 473 (78.2%) | 52 (8.6%) | 15 (2.5%) |
| **HPV can be passed on during sexual intercourse** | No | 488 | HPV Vaccine is Important | 49 (10.0%) | 358 (73.4%) | 45 (9.2%) | 36 (7.4%) |
| **Men can get HPV** | Yes | 437 | HPV Vaccine is Important | 40 (9.2%) | 341 (78.0%) | 43 (9.8%) | 13 (3.0%) |
| **Men can get HPV** | No | 656 | HPV Vaccine is Important | 74 (11.3%) | 490 (74.7%) | 54 (8.2%) | 38 (5.8%) |
| **A person could have HPV for many years without knowing it** | Yes | 951 | HPV Vaccine is Important | 97 (10.2%) | 730 (76.8%) | 87 (9.1%) | 37 (3.9%) |
| **A person could have HPV for many years without knowing it** | No | 142 | HPV Vaccine is Important | 17 (12.0%) | 101 (71.1%) | 10 (7.0%) | 14 (9.9%) |
| **The HPV vaccine is most effective if given to girls who have never had sex** | Yes | 807 | HPV Vaccine is Important | 82 (10.2%) | 634 (78.6%) | 65 (8.1%) | 26 (3.2%) |
| **The HPV vaccine is most effective if given to girls who have never had sex** | No | 286 | HPV Vaccine is Important | 32 (11.2%) | 197 (68.9%) | 32 (11.2%) | 25 (8.7%) |
| **The HPV vaccine offers protection against cervical cancer** | Yes | 1014 | HPV Vaccine is Important | 101 (10.0%) | 787 (77.6%) | 90 (8.9%) | 36 (3.6%) |
| **The HPV vaccine offers protection against cervical cancer** | No | 79 | HPV Vaccine is Important | 13 (16.5%) | 44 (55.7%) | 7 (8.9%) | 15 (19.0%) |
| **Girls who have had the HPV vaccine do not need VIA when they are older** | Yes | 239 | HPV Vaccine is Important | 22 (9.2%) | 186 (77.8%) | 24 (10.0%) | 7 (2.9%) |
| **Girls who have had the HPV vaccine do not need VIA when they are older** | No | 854 | HPV Vaccine is Important | 92 (10.8%) | 645 (75.5%) | 73 (8.5%) | 44 (5.2%) |
| **HPV can cause cervical cancer** | Yes | 703 | HPV Vaccine is Effective | 66 (9.4%) | 548 (78.0%) | 64 (9.1%) | 25 (3.6%) |
| **HPV can cause cervical cancer** | No | 390 | HPV Vaccine is Effective | 41 (10.5%) | 283 (72.6%) | 46 (11.8%) | 20 (5.1%) |
| **HPV can be passed on during sexual intercourse** | Yes | 605 | HPV Vaccine is Effective | 56 (9.3%) | 484 (80.0%) | 47 (7.8%) | 18 (3.0%) |
| **HPV can be passed on during sexual intercourse** | No | 488 | HPV Vaccine is Effective | 51 (10.5%) | 347 (71.1%) | 63 (12.9%) | 27 (5.5%) |
| **Men can get HPV** | Yes | 437 | HPV Vaccine is Effective | 41 (9.4%) | 345 (78.9%) | 37 (8.5%) | 14 (3.2%) |
| **Men can get HPV** | No | 656 | HPV Vaccine is Effective | 66 (10.1%) | 486 (74.1%) | 73 (11.1%) | 31 (4.7%) |
| **A person could have HPV for many years without knowing it** | Yes | 951 | HPV Vaccine is Effective | 90 (9.5%) | 731 (76.9%) | 98 (10.3%) | 32 (3.4%) |
| **A person could have HPV for many years without knowing it** | No | 142 | HPV Vaccine is Effective | 17 (12.0%) | 100 (70.4%) | 12 (8.5%) | 13 (9.2%) |
| **The HPV vaccine is most effective if given to girls who have never had sex** | Yes | 807 | HPV Vaccine is Effective | 78 (9.7%) | 637 (78.9%) | 68 (8.4%) | 24 (3.0%) |
| **The HPV vaccine is most effective if given to girls who have never had sex** | No | 286 | HPV Vaccine is Effective | 29 (10.1%) | 194 (67.8%) | 42 (14.7%) | 21 (7.3%) |
| **The HPV vaccine offers protection against cervical cancer** | Yes | 1014 | HPV Vaccine is Effective | 97 (9.6%) | 782 (77.1%) | 101 (10.0%) | 34 (3.4%) |
| **The HPV vaccine offers protection against cervical cancer** | No | 79 | HPV Vaccine is Effective | 10 (12.7%) | 49 (62.0%) | 9 (11.4%) | 11 (13.9%) |
| **Girls who have had the HPV vaccine do not need VIA when they are older** | Yes | 239 | HPV Vaccine is Effective | 23 (9.6%) | 185 (77.4%) | 27 (11.3%) | 4 (1.7%) |
| **Girls who have had the HPV vaccine do not need VIA when they are older** | No | 854 | HPV Vaccine is Effective | 84 (9.8%) | 646 (75.6%) | 83 (9.7%) | 41 (4.8%) |
| **HPV can cause cervical cancer** | Yes | 703 | Clinician Guidance | 64 (9.1%) | 571 (81.2%) | 61 (8.7%) | 7 (1.0%) |
| **HPV can cause cervical cancer** | No | 390 | Clinician Guidance | 33 (8.5%) | 313 (80.3%) | 35 (9.0%) | 9 (2.3%) |
| **HPV can be passed on during sexual intercourse** | Yes | 605 | Clinician Guidance | 53 (8.8%) | 497 (82.1%) | 48 (7.9%) | 7 (1.2%) |
| **HPV can be passed on during sexual intercourse** | No | 488 | Clinician Guidance | 44 (9.0%) | 387 (79.3%) | 48 (9.8%) | 9 (1.8%) |
| **Men can get HPV** | Yes | 437 | Clinician Guidance | 37 (8.5%) | 352 (80.5%) | 42 (9.6%) | 6 (1.4%) |
| **Men can get HPV** | No | 656 | Clinician Guidance | 60 (9.1%) | 532 (81.1%) | 54 (8.2%) | 10 (1.5%) |
| **A person could have HPV for many years without knowing it** | Yes | 951 | Clinician Guidance | 82 (8.6%) | 774 (81.4%) | 80 (8.4%) | 15 (1.6%) |
| **A person could have HPV for many years without knowing it** | No | 142 | Clinician Guidance | 15 (10.6%) | 110 (77.5%) | 16 (11.3%) | 1 (0.7%) |
| **The HPV vaccine is most effective if given to girls who have never had sex** | Yes | 807 | Clinician Guidance | 68 (8.4%) | 667 (82.7%) | 63 (7.8%) | 9 (1.1%) |
| **The HPV vaccine is most effective if given to girls who have never had sex** | No | 286 | Clinician Guidance | 29 (10.1%) | 217 (75.9%) | 33 (11.5%) | 7 (2.4%) |
| **The HPV vaccine offers protection against cervical cancer** | Yes | 1014 | Clinician Guidance | 90 (8.9%) | 824 (81.3%) | 88 (8.7%) | 12 (1.2%) |
| **The HPV vaccine offers protection against cervical cancer** | No | 79 | Clinician Guidance | 7 (8.9%) | 60 (75.9%) | 8 (10.1%) | 4 (5.1%) |
| **Girls who have had the HPV vaccine do not need VIA when they are older** | Yes | 239 | Clinician Guidance | 22 (9.2%) | 191 (79.9%) | 24 (10.0%) | 2 (0.8%) |
| **Girls who have had the HPV vaccine do not need VIA when they are older** | No | 854 | Clinician Guidance | 75 (8.8%) | 693 (81.1%) | 72 (8.4%) | 14 (1.6%) |
| **HPV can cause cervical cancer** | Yes | 703 | Moderate Side Effects | 209 (29.7%) | 255 (36.3%) | 187 (26.6%) | 52 (7.4%) |
| **HPV can cause cervical cancer** | No | 390 | Moderate Side Effects | 128 (32.8%) | 101 (25.9%) | 113 (29.0%) | 48 (12.3%) |
| **HPV can be passed on during sexual intercourse** | Yes | 605 | Moderate Side Effects | 187 (30.9%) | 211 (34.9%) | 160 (26.4%) | 47 (7.8%) |
| **HPV can be passed on during sexual intercourse** | No | 488 | Moderate Side Effects | 150 (30.7%) | 145 (29.7%) | 140 (28.7%) | 53 (10.9%) |
| **Men can get HPV** | Yes | 437 | Moderate Side Effects | 129 (29.5%) | 152 (34.8%) | 128 (29.3%) | 28 (6.4%) |
| **Men can get HPV** | No | 656 | Moderate Side Effects | 208 (31.7%) | 204 (31.1%) | 172 (26.2%) | 72 (11.0%) |
| **A person could have HPV for many years without knowing it** | Yes | 951 | Moderate Side Effects | 295 (31.0%) | 317 (33.3%) | 263 (27.7%) | 76 (8.0%) |
| **A person could have HPV for many years without knowing it** | No | 142 | Moderate Side Effects | 42 (29.6%) | 39 (27.5%) | 37 (26.1%) | 24 (16.9%) |
| **The HPV vaccine is most effective if given to girls who have never had sex** | Yes | 807 | Moderate Side Effects | 244 (30.2%) | 275 (34.1%) | 232 (28.7%) | 56 (6.9%) |
| **The HPV vaccine is most effective if given to girls who have never had sex** | No | 286 | Moderate Side Effects | 93 (32.5%) | 81 (28.3%) | 68 (23.8%) | 44 (15.4%) |
| **The HPV vaccine offers protection against cervical cancer** | Yes | 1014 | Moderate Side Effects | 311 (30.7%) | 334 (32.9%) | 285 (28.1%) | 84 (8.3%) |
| **The HPV vaccine offers protection against cervical cancer** | No | 79 | Moderate Side Effects | 26 (32.9%) | 22 (27.8%) | 15 (19.0%) | 16 (20.3%) |
| **Girls who have had the HPV vaccine do not need VIA when they are older** | Yes | 239 | Moderate Side Effects | 68 (28.5%) | 86 (36.0%) | 69 (28.9%) | 16 (6.7%) |
| **Girls who have had the HPV vaccine do not need VIA when they are older** | No | 854 | Moderate Side Effects | 269 (31.5%) | 270 (31.6%) | 231 (27.0%) | 84 (9.8%) |
| **HPV can cause cervical cancer** | Yes | 703 | Serious Side Effects | 211 (30.0%) | 247 (35.1%) | 190 (27.0%) | 55 (7.8%) |
| **HPV can cause cervical cancer** | No | 390 | Serious Side Effects | 123 (31.5%) | 105 (26.9%) | 114 (29.2%) | 48 (12.3%) |
| **HPV can be passed on during sexual intercourse** | Yes | 605 | Serious Side Effects | 188 (31.1%) | 205 (33.9%) | 164 (27.1%) | 48 (7.9%) |
| **HPV can be passed on during sexual intercourse** | No | 488 | Serious Side Effects | 146 (29.9%) | 147 (30.1%) | 140 (28.7%) | 55 (11.3%) |
| **Men can get HPV** | Yes | 437 | Serious Side Effects | 123 (28.1%) | 155 (35.5%) | 128 (29.3%) | 31 (7.1%) |
| **Men can get HPV** | No | 656 | Serious Side Effects | 211 (32.2%) | 197 (30.0%) | 176 (26.8%) | 72 (11.0%) |
| **A person could have HPV for many years without knowing it** | Yes | 951 | Serious Side Effects | 286 (30.1%) | 319 (33.5%) | 265 (27.9%) | 81 (8.5%) |
| **A person could have HPV for many years without knowing it** | No | 142 | Serious Side Effects | 48 (33.8%) | 33 (23.2%) | 39 (27.5%) | 22 (15.5%) |
| **The HPV vaccine is most effective if given to girls who have never had sex** | Yes | 807 | Serious Side Effects | 246 (30.5%) | 271 (33.6%) | 234 (29.0%) | 56 (6.9%) |
| **The HPV vaccine is most effective if given to girls who have never had sex** | No | 286 | Serious Side Effects | 88 (30.8%) | 81 (28.3%) | 70 (24.5%) | 47 (16.4%) |
| **The HPV vaccine offers protection against cervical cancer** | Yes | 1014 | Serious Side Effects | 313 (30.9%) | 329 (32.4%) | 285 (28.1%) | 87 (8.6%) |
| **The HPV vaccine offers protection against cervical cancer** | No | 79 | Serious Side Effects | 21 (26.6%) | 23 (29.1%) | 19 (24.1%) | 16 (20.3%) |
| **Girls who have had the HPV vaccine do not need VIA when they are older** | Yes | 239 | Serious Side Effects | 69 (28.9%) | 83 (34.7%) | 70 (29.3%) | 17 (7.1%) |
| **Girls who have had the HPV vaccine do not need VIA when they are older** | No | 854 | Serious Side Effects | 265 (31.0%) | 269 (31.5%) | 234 (27.4%) | 86 (10.1%) |
| **HPV can cause cervical cancer** | Yes | 703 | Long-term Side Effects | 214 (30.4%) | 248 (35.3%) | 186 (26.5%) | 55 (7.8%) |
| **HPV can cause cervical cancer** | No | 390 | Long-term Side Effects | 118 (30.3%) | 114 (29.2%) | 107 (27.4%) | 51 (13.1%) |
| **HPV can be passed on during sexual intercourse** | Yes | 605 | Long-term Side Effects | 188 (31.1%) | 206 (34.0%) | 163 (26.9%) | 48 (7.9%) |
| **HPV can be passed on during sexual intercourse** | No | 488 | Long-term Side Effects | 144 (29.5%) | 156 (32.0%) | 130 (26.6%) | 58 (11.9%) |
| **Men can get HPV** | Yes | 437 | Long-term Side Effects | 131 (30.0%) | 149 (34.1%) | 129 (29.5%) | 28 (6.4%) |
| **Men can get HPV** | No | 656 | Long-term Side Effects | 201 (30.6%) | 213 (32.5%) | 164 (25.0%) | 78 (11.9%) |
| **A person could have HPV for many years without knowing it** | Yes | 951 | Long-term Side Effects | 294 (30.9%) | 321 (33.8%) | 255 (26.8%) | 81 (8.5%) |
| **A person could have HPV for many years without knowing it** | No | 142 | Long-term Side Effects | 38 (26.8%) | 41 (28.9%) | 38 (26.8%) | 25 (17.6%) |
| **The HPV vaccine is most effective if given to girls who have never had sex** | Yes | 807 | Long-term Side Effects | 249 (30.9%) | 276 (34.2%) | 222 (27.5%) | 60 (7.4%) |
| **The HPV vaccine is most effective if given to girls who have never had sex** | No | 286 | Long-term Side Effects | 83 (29.0%) | 86 (30.1%) | 71 (24.8%) | 46 (16.1%) |
| **The HPV vaccine offers protection against cervical cancer** | Yes | 1014 | Long-term Side Effects | 315 (31.1%) | 337 (33.2%) | 275 (27.1%) | 87 (8.6%) |
| **The HPV vaccine offers protection against cervical cancer** | No | 79 | Long-term Side Effects | 17 (21.5%) | 25 (31.6%) | 18 (22.8%) | 19 (24.1%) |
| **Girls who have had the HPV vaccine do not need VIA when they are older** | Yes | 239 | Long-term Side Effects | 70 (29.3%) | 86 (36.0%) | 65 (27.2%) | 18 (7.5%) |
| **Girls who have had the HPV vaccine do not need VIA when they are older** | No | 854 | Long-term Side Effects | 262 (30.7%) | 276 (32.3%) | 228 (26.7%) | 88 (10.3%) |
| **HPV can cause cervical cancer** | Yes | 703 | HPV Vaccine is Risky | 134 (19.1%) | 235 (33.4%) | 184 (26.2%) | 150 (21.3%) |
| **HPV can cause cervical cancer** | No | 390 | HPV Vaccine is Risky | 73 (18.7%) | 115 (29.5%) | 89 (22.8%) | 113 (29.0%) |
| **HPV can be passed on during sexual intercourse** | Yes | 605 | HPV Vaccine is Risky | 129 (21.3%) | 205 (33.9%) | 155 (25.6%) | 116 (19.2%) |
| **HPV can be passed on during sexual intercourse** | No | 488 | HPV Vaccine is Risky | 78 (16.0%) | 145 (29.7%) | 118 (24.2%) | 147 (30.1%) |
| **Men can get HPV** | Yes | 437 | HPV Vaccine is Risky | 87 (19.9%) | 128 (29.3%) | 128 (29.3%) | 94 (21.5%) |
| **Men can get HPV** | No | 656 | HPV Vaccine is Risky | 120 (18.3%) | 222 (33.8%) | 145 (22.1%) | 169 (25.8%) |
| **A person could have HPV for many years without knowing it** | Yes | 951 | HPV Vaccine is Risky | 186 (19.6%) | 306 (32.2%) | 245 (25.8%) | 214 (22.5%) |
| **A person could have HPV for many years without knowing it** | No | 142 | HPV Vaccine is Risky | 21 (14.8%) | 44 (31.0%) | 28 (19.7%) | 49 (34.5%) |
| **The HPV vaccine is most effective if given to girls who have never had sex** | Yes | 807 | HPV Vaccine is Risky | 149 (18.5%) | 261 (32.3%) | 222 (27.5%) | 175 (21.7%) |
| **The HPV vaccine is most effective if given to girls who have never had sex** | No | 286 | HPV Vaccine is Risky | 58 (20.3%) | 89 (31.1%) | 51 (17.8%) | 88 (30.8%) |
| **The HPV vaccine offers protection against cervical cancer** | Yes | 1014 | HPV Vaccine is Risky | 192 (18.9%) | 331 (32.6%) | 262 (25.8%) | 229 (22.6%) |
| **The HPV vaccine offers protection against cervical cancer** | No | 79 | HPV Vaccine is Risky | 15 (19.0%) | 19 (24.1%) | 11 (13.9%) | 34 (43.0%) |
| **Girls who have had the HPV vaccine do not need VIA when they are older** | Yes | 239 | HPV Vaccine is Risky | 55 (23.0%) | 75 (31.4%) | 67 (28.0%) | 42 (17.6%) |
| **Girls who have had the HPV vaccine do not need VIA when they are older** | No | 854 | HPV Vaccine is Risky | 152 (17.8%) | 275 (32.2%) | 206 (24.1%) | 221 (25.9%) |
