# Supplementary material for: Changes in attitudes toward the HPV vaccine in Kenya
Source: PLOS Glob Public Health. 2026 Oct 23;6(10):e0007508. doi: 10.1371/journal.pgph.0007508 (PMC13693870; doi:10.1371/journal.pgph.0007508)
Supplement: Supplementary file 7 [file pgph.0007508.s007.docx]

S7 Table: Transitions to and from “I don’t know” responses between 2022 and 2024, with percentages calculated within the “I don’t know” population at each time point.

| Question | Total DK in 2022 | Positive in 2024 | Negative in 2024 | DK in 2024 | Total DK in 2024 | Positive in 2022 | Negative in 2022 | DK in 2022 |
| --- | --- | --- | --- | --- | --- | --- | --- | --- |
| The HPV vaccine is important for my daughter’s health | 33 | 29 (87.9%) | 2 (6.1%) | 2 (6.1%) | 20 | 17 (85.0%) | 1 (5.0%) | 2 (10.0%) |
| Getting the HPV vaccine is a good way to protect my daughter from disease | 28 | 26 (92.9%) | 0 (0.0%) | 2 (7.1%) | 19 | 16 (84.2%) | 1 (5.3%) | 2 (10.5%) |
| I do what the clinician recommends about HPV vaccine | 7 | 7 (100.0%) | 0 (0.0%) | 0 (0.0%) | 9 | 8 (88.9%) | 1 (11.1%) | 0 (0.0%) |
| The HPV vaccine carries more risks than older vaccines | 211 | 131 (62.1%) | 52 (24.6%) | 28 (13.3%) | 80 | 35 (43.8%) | 17 (21.2%) | 28 (35.0%) |
| I am concerned about mild to moderate short-term effects of HPV vaccine | 63 | 22 (34.9%) | 34 (54.0%) | 7 (11.1%) | 44 | 16 (36.4%) | 21 (47.7%) | 7 (15.9%) |
| I am concerned about serious short-term effects of HPV vaccine | 67 | 29 (43.3%) | 29 (43.3%) | 9 (13.4%) | 45 | 16 (35.6%) | 20 (44.4%) | 9 (20.0%) |
| I am concerned about long-term effects of HPV vaccine | 69 | 33 (47.8%) | 31 (44.9%) | 5 (7.2%) | 42 | 18 (42.9%) | 19 (45.2%) | 5 (11.9%) |
