# Supplementary material for: Changes in attitudes toward the HPV vaccine in Kenya
Source: PLOS Glob Public Health. 2026 Oct 23;6(10):e0007508. doi: 10.1371/journal.pgph.0007508 (PMC13693870; doi:10.1371/journal.pgph.0007508)
Supplement: Supplementary file 8 [file pgph.0007508.s008.docx]

S8 Table: Odds ratios from multivariate logistic regression analyses. Values greater than 1 indicate higher odds of a positive attitude change, whereas values less than 1 indicates higher odds of a negative attitude change. Statistically significant estimates (p<0.05) are marked with an asterisk.

| Variable | Category | HPV Vaccine is Important | HPV Vaccine is Effective | HPV Vaccine is Risky | Moderate Side Effects | Serious Side Effects | Long-term Side Effects | Clinician Guidance |
| --- | --- | --- | --- | --- | --- | --- | --- | --- |
| Gender | Female (ref) | 1 | 1 | 1 | 1 | 1 | 1 | 1 |
|  | Male | 1.03 | 0.8 | 1.03 | 0.92 | 0.89 | 0.96 | 0.82 |
| Education Level | No school | 0.55 | 1.02 | 0.56 | 0.91 | 0.73 | 0.73 | 0.72 |
|  | Primary | 0.79 | 0.91 | 1.02 | 0.71 | 0.72 | 0.81 | 1.3 |
|  | Secondary | 0.98 | 1.13 | 0.81 | 0.94 | 1.03 | 1.17 | 1.37 |
|  | Higher (ref) | 1 | 1 | 1 | 1 | 1 | 1 | 1 |
| Residence Area | Rural (ref) | 1 | 1 | 1 | 1 | 1 | 1 | 1 |
|  | Town | 1.06 | 0.83 | 1.28 | 1.11 | 0.96 | 1.11 | 1.66 (*) |
|  | Urban | 1.04 | 0.79 | 1.42 (*) | 1.2 | 0.83 | 1.12 | 0.93 |
| Religion | Anglican | 1.15 | 0.91 | 0.97 | 0.79 | 0.96 | 1.18 | 0.72 |
|  | Catholic | 1.05 | 1.17 | 1.1 | 0.8 | 1 | 1.05 | 1.03 |
|  | Muslim | 0.82 | 0.98 | 0.7 | 1.33 | 1.28 | 1.16 | 0.61 |
|  | No Religion | 0.38 | 1.76 | 0.76 | 0.41 | 0.4 | 0.45 | 1.56 |
|  | Protestant/ Pentecostal (ref) | 1 | 1 | 1 | 1 | 1 | 1 | 1 |
|  | SDA | 2.1 (*) | 1.99 (*) | 0.98 | 1.06 | 1.12 | 1.04 | 1.17 |
|  | Traditional/Tribal | 1.15 | 1.56 | 0.99 | 0.65 | 0.59 | 0.41 (*) | 1.3 |
| Marital Status | Previously Married | 1.22 | 0.96 | 0.72 | 1.31 | 1.2 | 1.06 | 0.6 |
|  | Partnered/Married (ref) | 1 | 1 | 1 | 1 | 1 | 1 | 1 |
|  | Single | 1.23 | 0.88 | 1.1 | 1.18 | 0.88 | 0.81 | 1.1 |
| Employment Status | Formal Employment | 0.78 | 1.17 | 1.28 | 1.04 | 1.05 | 0.94 | 0.99 |
|  | Self-Employed/Informal (ref) | 1 | 1 | 1 | 1 | 1 | 1 | 1 |
|  | Unemployed | 0.81 | 1.05 | 1.12 | 0.95 | 1.31 | 0.95 | 1.14 |
| Household Income | High | 0.85 | 0.63 | 0.97 | 0.96 | 0.86 | 0.84 | 1.25 |
|  | Mid | 1.24 | 1.23 | 0.95 | 0.89 | 0.8 | 0.92 | 1.07 |
|  | Low (ref) | 1 | 1 | 1 | 1 | 1 | 1 | 1 |
| HPV Knowledge Level | Low | 1.38 | 1.02 | 1.14 | 1.22 | 1.3 | 1.16 | 1.11 |
|  | Medium | 1.36 | 1.11 | 1.11 | 1.05 | 1.15 | 0.87 | 1.13 |
|  | High (ref) | 1 | 1 | 1 | 1 | 1 | 1 | 1 |
